# Light-controlled Spo11-less meiotic DNA breaks by MagTAQing lead to chromosomal aberrations

**DOI:** 10.1093/nar/gkaf206

**Published:** 2025-04-10

**Authors:** Hideyuki Yone, Yuri Kawashima, Hayato Hirai, Arisa H Oda, Moritoshi Sato, Hiromitsu Kono, Kunihiro Ohta

**Affiliations:** Department of Life Sciences, Graduate School of Arts and Sciences, The University of Tokyo, Komaba 3-8-1, Meguro-ku, Tokyo 153-8902, Japan; Department of Life Sciences, Graduate School of Arts and Sciences, The University of Tokyo, Komaba 3-8-1, Meguro-ku, Tokyo 153-8902, Japan; Department of Life Sciences, Graduate School of Arts and Sciences, The University of Tokyo, Komaba 3-8-1, Meguro-ku, Tokyo 153-8902, Japan; Department of Life Sciences, Graduate School of Arts and Sciences, The University of Tokyo, Komaba 3-8-1, Meguro-ku, Tokyo 153-8902, Japan; Department of Life Sciences, Graduate School of Arts and Sciences, The University of Tokyo, Komaba 3-8-1, Meguro-ku, Tokyo 153-8902, Japan; Kanagawa Institute of Industrial Science and Technology (KISTEC), 3-2-1 Sakado, Takatsu-ku, Kawasaki, Kanagawa 213-0012, Japan; Department of Life Sciences, Graduate School of Arts and Sciences, The University of Tokyo, Komaba 3-8-1, Meguro-ku, Tokyo 153-8902, Japan; Department of Life Sciences, Graduate School of Arts and Sciences, The University of Tokyo, Komaba 3-8-1, Meguro-ku, Tokyo 153-8902, Japan; Universal Biology Institute, The University of Tokyo, Hongo 7-3-1, Bunkyo-Ku, Tokyo 113-0033, Japan; Collaborative Research Institute for Innovative Microbiology, The University of Tokyo, Hongo 7-3-1, Bunkyo-Ku, Tokyo 113-0033, Japan

## Abstract

TAQing technologies are based on the restriction enzyme-induced DNA double-strand break (DSB) formation in living cells, which results in large-scale genomic rearrangements and phenotypic alterations. Originally, the TAQing system requires heat treatments to activate restriction enzymes, which sometimes leads to cell toxicity or stress responses. Here, we developed a blue-light-controlled MagTAQing system, which induces DSBs exclusively upon blue-light exposure by assembling the split restriction enzymes via Magnet modules. Application of MagTAQing to mitotic budding yeast cells successfully triggered various genomic rearrangements upon blue-light exposure. Since this technology enables the conditional induction of genomic rearrangements in specific cells or tissues, we employed MagTAQing on meiotic yeast cells lacking the recombinase Spo11 to induce artificial DSBs. Consequently, Spo11-independent meiotic DSBs resulted in aneuploidies and nonallelic homologous recombinations between repetitive sequences such as ribosomal DNA and retrotransposons. These results suggest a pivotal role of Spo11-induced recombination in preventing chromosomal abnormality.

## Graphical Abstract

**Graphical Abstract:**
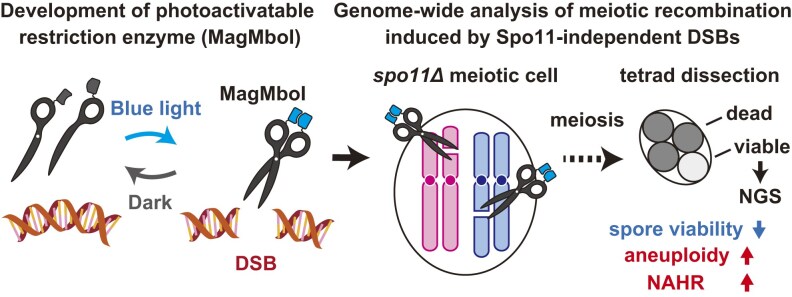

## Introduction

DNA double-strand breaks (DSBs) that occur in mitotic cells can induce chromosomal translocations (TLs), deletions (DELs), loss of heterozygosity (LOH) events, duplications (DUPs), and increasing genomic instability. On the other hand, DSBs during meiosis are formed only at limited sites called DSB hotspots during prophase I through complex mechanisms involving various meiosis-specific recombinases, including the DSB-forming protein Spo11. Moreover, recombinations induced during meiosis are mostly homologous recombinations (HRs) between homologous chromosomes, which are categorized into crossovers (COs) and noncrossovers (NCOs) [1–3]. In meiosis, TLs between different chromosomes and non-HRs between repetitive sequences such as ribosomal DNA (rDNA) and retrotransposable elements are suppressed at very low frequencies [4–6]. This maintains the stable composition of chromosomes in the gametes and is believed to prevent the occurrence of chromosomal abnormalities.

When artificial DSBs are introduced in mitotic cells, it has been reported that HR and nonhomologous end joining (NHEJ) occur at the DSB ends [7]. We developed a “TAQing system” that introduces a restriction enzyme TaqI into living cells and induces DSBs at multiple sites conditionally through heat activation of the restriction enzyme [8]. Applying the TAQing system to eukaryotic (fungi and plant) cells induces a variety of chromosomal structural changes, such as TLs, DELs, DUPs, break-induced replications (BIRs), and aneuploidy formation, along with accompanying phenotypic changes [8–11].

Meiotic DSBs are generated by Spo11, a type II topoisomerase-like transesterase, which requires accessory proteins that function in cooperation with Spo11 to catalyze DSBs [12, 13]. Spo11 can form concerted double DSBs with double-stranded gaps which require subsequent gap repair HR [14, 15]. Moreover, DSB formation by Spo11 is governed by complex mechanisms, such as the phosphorylation of Spo11 accessory proteins in couple with the meiotic cell cycle [16–21]. In addition, DSB formation is regulated by a combination of multiple factors such as the three-dimensional chromosomal structures including axis-loop structures [6, 22–28], local chromatin structures [29, 30], and histone modifications [31–34].

In nematodes and fission yeast lacking Spo11, meiosis can be rescued by supplying artificial DSBs through radiation exposure or introducing DNA break-inducing mutations [35–37]. Thus, in these organisms which have relatively small number of chromosomes with pairing centers, it seems that Spo11-dependent DSBs are dispensable for the formation of functional gametes. However, in most species, a Spo11-dependent DSB formation controlled by complex regulatory mechanisms is indispensable [38]. The reason for this has not been fully elucidated yet.

In this study, we developed a restriction enzyme that can be activated by blue light and successfully induced mitotic and meiotic DSBs in budding yeast cells with minimal invasiveness. Previous studies on meiotic recombination have introduced Spo11-independent DSBs at single loci, such as HO and VDE recognition sites [39–41]. However, our system is introducing hundreds of DSBs simultaneously across the genome and observe recombination in a manner similar to Spo11. As previously reported [8], numerous NHEJ-mediated chromosomal rearrangements were generated during mitosis. In contrast, the introduction of artificial DSBs during meiosis caused many HRs, while there were substantial numbers of TLs and noncanonical recombinations between repetitive sequences, such as transposons and rDNA, resulting in many inviable spores. These results suggest that in budding yeast, artificial induction of DSBs is not enough to rescue meiosis properly, and complex mechanisms associated with Spo11 might be suppressing chromosomal abnormalities and aneuploidy formation.

## Materials and methods

### Yeast strains and culture

The diploid *Saccharomyces cerevisiae* strain SYW4 for mitotic MagTAQing was constructed by cell fusion of strains S288C-derived haploid (YPH499; *MATa ura3-52 lys2-801 ade2-101 trpl-Δ63 his3-Δ200 leu2-Δ1*) and the SK1-derived haploid (S799 *flo8Δ*; *MATa ura3 lys2 ho::LYS2 leu2Δ arg4-bgl cyh2-z flo8:hyg*). The Spo11-less diploid hybrid strain (YTG14 *spo11Δ*; *Mata/α LYS2/lys2 ho::LYS2/LYS2 leu2/leu2 (asp718-ecoRi) Arg4/arg4-bgl CYH2/cyh2-z spo11Δ::URA3/spo11Δ::URA3*) constructed in a previous study [42] was used for meiotic MagTAQing. Plasmid DNA transformation was performed using the LiAc/SS carrier DNA/PEG method [43]. Yeast cells were cultured at 30°C in yeast extract–peptone–dextrose (YPD; 10 g/l yeast extract, 20 g/l peptone, and 20 g/l dextrose) or synthetic defined/monosodium glutamic acid without leucine (SD/MSG-Leu; 1.7 g/l yeast nitrogen base without amino acids, 0.69 g/l CSM-Leu, 1.0 g/l L-glutamic acid monosodium salt hydrate, and 20 g/l dextrose). In meiotic MagTAQing experiments, pre-sporulation medium (SPS; 5 g/l yeast extract, 10 g/l peptone, 1.7 g/l yeast nitrogen base without ammonium sulfate and amino acids, 50 mM potassium phthalate, 10 g/l potassium acetate, 5 g/l ammonium sulfate, 0.77 g/l complete supplement mixture, and 0.001% polyethylene glycol, pH 5.5) and sporulation medium (SPM; 10 g/l potassium acetate, 154 mg/l complete supplement mixture, and 0.001% polyethylene glycol) were used for meiotic induction. Strains used in this study were listed in Supplementary Table S1.

### Construction of photoactivatable restriction enzyme

The DNA sequence of MboI from *Moraxella bovis* ATCC10900 was codon optimized for budding yeast. The MboI sequence was split into the N-terminal (N-MboI) and C-terminal (C-MboI) fragments, fused with nMag and pMag, respectively. To determine the optimal split sites, we constructed MboI fragments with 15 different split sites. A nuclear localization sequence (NLS) was added to the N-terminus of N-MboI and C-MboI, and these two fragments were fused with a 2A self-cleavage peptide (P2A). The promoter sequence of the *CUP1* gene was inserted upstream of the *MagMboI* gene to conditionally induce expression with Cu^2+^. These DNA fragments were cloned into pRS415 and pORF-CLONE vectors using In-Fusion Snap Assembly Master Mix (Takara Bio Inc., Japan).

### Blue light

The blue light used in all experiments was a blue LED (465–475 nm, 2–3 mW/cm², NSPB510AS, Nichia Corporation, Japan) incorporated into a USB-powered push-touch light block (4972822310628, Daiso Industries Co., Ltd., Japan). The distance from the light source was 30 cm.

### Spot assay

The haploid strain S288C harboring the empty vector (pRS415) or respective MagMboI variant vector was cultured at 30°C in SD/MSG-Leu. Ten-fold serial dilutions of culture were spotted onto SD/MSG-Leu agar plates. Plates were incubated at 30°C for 48 h under dark conditions or blue light irradiation (470 nm).

### Quantification of the fraction of cleaved DNA

The samples were analyzed using capillary electrophoresis by Agilent 2200 TapeStation system (Agilent, Santa Clara, CA, USA). TapeStation was performed using genomic DNA screen tapes for analyzing the cleaved DNA. The samples were prepared according to the manufacturer’s protocol. TapeStation software was used to quantify the cleaved DNA. The cleaved region was defined from 200 to 13 000 bp.

### Western blotting

The empty vector (pRS415) or the MagMboI-8 vector was introduced into YPH499 cells harboring *RAD53* gene with a C-terminal 3 × FLAG tag [8]. The cells were cultured in SD/MSG-Leu medium added with 150 μM CuSO_4_ for 1 h in the dark and exposed to blue light for 30 min. After light irradiation, cells were cultured for 12 h in the dark. The cultured cells were washed twice with phosphate-buffered saline (PBS; 137 mM NaCl, 2.7 mM KCl, 1.8 mM KH_2_PO_4_, 10 mM Na_2_HPO_4_, pH 7.4) and then frozen in liquid nitrogen. The frozen cells were disrupted with 0.5 mm zirconia beads in lysis buffer (60 mM β-glycerophosphate, 1 mM dithiothreitol, 1 mM phenylmethylsulfonyl fluoride, 0.1 mM sodium orthovanadate, 15 mM p-nitrophenyl phosphate, cOmplete Tablets, Mini ethylenediaminetetraacetic acid-free, EASYpack (04693159001, Roche, Switzerland) using a Multi-Beads Shocker (YASUI KIKAI, Japan). The protein concentration of lysate was measured using the Bradford assay. The lysate, added with sodium dodecyl sulfate buffer (193-11032, FUJIFILM Wako Pure Chemical Corporation, Japan), were boiled at 100°C for 3 min. The denatured sample was then electrophoresed in 10% polyacrylamide gel (Mini-PROTEAN TGX Gels, 4561036, Bio-Rad Laboratories, Inc., USA) and transferred to a polyvinylidene difluoride membrane (IPVH304F0, Merck Millipore, USA). Anti-FLAG (M2 F1804; Sigma–Aldrich, St. Louis, USA; dilution, 1:1000) and anti-mouse IgG-HRP (NA931-1ML; GE Healthcare, USA; dilution, 1:2000) antibodies were used for immunoblotting. The images were acquired by ImageQuant^TM^ LAS4000mini (Cytiva, Japan). The signal intensity of gel bands was quantified with ImageJ.

### Protein structure prediction with AlphaFold2

We generated the 3D structural models of MboI and MagMboI-8 proteins using AlphaFold (v2.1.1) [44]. The databases were downloaded on 2 December 2021. The program was run with the following parameters “–max_template_date = 2021–12-01 –model_ preset = monomer –db_preset = full_dbs –is_prokaryote_list = false”. The pdb files from each highest-ranking structural model were visualized with the PyMOL Molecular Graphics System (version 3.0, Schrödinger, Inc., USA).

### MagTAQing for mitotic cells

The fused strain SYW4 harboring the pRS415-MagMboI-8 vector was cultured overnight in SD/MSG-Leu medium at 30°C and diluted into fresh media. Then, 150 μM CuSO_4_ was added and cultured for 4 h to induce the *MagMboI-8* gene expression. Cultured cells in the exponential growth phase were washed with distilled water and then exposed to blue light (470 nm) for 30 min. After light irradiation, cells were plated on YPD agar plates and incubated at 30°C for at least 5 days in the dark for single colony formation. Of >1000 colonies, we isolated 19 of MagTAQed mutants with altered cell morphology (e.g. cell size, roundness, and colony morphology). The images of the MagTAQed cells cultured in liquid YPD media at 30°C were acquired using microscopy BZ-X810 (Keyence Corporation, Japan) equipped with Plan Apochromat 100× Oil lens (BZ-PA100, Keyence Corporation, Japan).

### Illumina short-read sequencing

Genomic DNA extraction was performed by the Dr GenTLE™ High Recovery for Yeast Kit (Takara Bio Inc., Japan). Covaris Focused-ultrasonicator M220 (Covaris, LLC., USA) was used for DNA shearing to a size of ∼300 bp. DNA library was prepared using the NEBNext Ultra II DNA Library Prep Kit for Illumina (New England Biolabs, USA) and NEBNext Multiplex Oligos for Illumina (New England Biolabs, USA). DNA sequencing was performed by Illumina HiSeq X Ten (Illumina, Inc., USA).

### Mutation detection pipeline for diploid mutants

The workflow for mutation detection is illustrated in Supplementary Fig. S1. First, sequence reads were mapped to the reference genome sequences of the diploid strain (SYW4), parental haploid strains SK1 (S799), and S288C (YPH499 or YKN1419) using a Burrows–Wheeler Aligner (BWA) [45], as described in previous studies [8, 11]. Next, small variants such as single nucleotide variations (SNVs) and InDels were detected using the Genome Analysis Toolkit (GATK) [46]. LOHs such as SGCs and BIRs were identified based on mapped coverage and allele frequency of variants. When sequence reads are mapped to the diploid genome, the LOH tracts show reciprocal alterations in coverage on homologous chromosomes. On the other hand, when mapping to the parental haploid genomes, variants were detected at high allele frequencies only on the respective parental chromosomes. LOH tracts extending to the end of a chromosome was defined as BIRs, while interstitial LOH tracts were defined as SGCs. TLs were classified into two types by their breakpoint features; NHEJ-mediated translocations (NMTLs) with chimeric reads and discordant paired-end reads from different chromosome locus, and nonallelic homologous recombinations (NAHRs) as TLs at homologous repetitive sequences. NMTLs were identified by calling chimeric alignments with SAMtools [47] and finding “discordant pair-end reads” around breakpoints with marked changes in mapped depth, as described in previous studies [8, 11]. NAHRs were detected by examining chimeric reads which included repetitive sequences with marked changes in mapped coverage and searching for other homogenous repetitive sequences with the same changes in mapped coverage. All NMTLs and NAHRs were confirmed by PCR (Primers were listed in Supplementary Table S2). Circular plots visualizing genome rearrangements were generated by OMGenomics Circa 1.2.2 (https://omgenomics.com/circa/). Aneuploidy was detected based on changes in coverage and allele frequency using the Integrated Genomics Viewer (IGV) [48]. To exclude false-positive mutations, identical mutations detected in the control strains were eliminated. Structural variations of MagTAQed isolates and MagTAQed RTG isolates were listed in Supplementary Tables S3 and S4, respectively.

### MagTAQing for meiotic cells

The diploid hybrid strain YTG14 *spo11Δ* was used for meiotic MagTAQing to exclude the influence of the endogenous meiotic DSB [42]. YTG14 *spo11Δ* harboring the pORF-CLONE-MagMboI-8 vector was cultured in SPM medium added with 150 μM CuSO_4_. After 3.5 h of culture in SPM medium, cells were exposed to blue light (470 nm) for 30 min by illuminating flasks during the culture, followed by incubation under dark conditions for 20 h. Viable spores were dissected with MSM400 (Singer Instruments, UK). In RTG experiments, cells exposed to blue light were rapidly plated on YPD agar plates. After two rounds of single colony isolations, we obtained MagTAQed RTG isolates with altered cellular morphologies, as in the mitotic MagTAQing experiments.

### Random spore analysis

One microliter of SPM culture medium was washed twice with sterile water and resuspended in Zymolyase solution (0.5 mg/ml in 1 M sorbitol). The suspension was incubated at 30°C for 30 min, then placed on ice. The total volume was adjusted to 1 ml, and the appropriate dilution was prepared. The diluted suspension was plated onto YPD plates, and colony numbers were counted to calculate spore viability.

### Mutation detection pipeline for haploid spores

The workflow for mutation detection is illustrated in Supplementary Fig. S2. First, genomic analysis of wild-type *SPO11^+^* spores obtained in a previous study [42] was performed. CO and NCO in *SPO11^+^* spores were detected by RecombineX [49] which is optimized for four viable spore tetrads and euploid spores, as described in the previous study [42]. A total of 1383 COs and 1969 NCOs were detected in 72 *SPO11^+^* spores dissected from 18 tetrads (Supplementary Table S5). Of 1969 NCOs detected in *SPO11^+^* spores, ∼95% of NCOs in *SPO11^+^* spores were <5 kb in length (Supplementary Fig. S2). A single NAHR event was also detected using ReconbineX [49] and IGV [48] (Supplementary Table S5). Next, mapping of sequence reads of MagTAQed spores to the reference genome sequences of the diploid strain (YTG14) and haploid parent strains SK1 (S799), and S288C (YKN1419) were performed using a BWA [45]. Small-scale mutations (SNVs and InDels) with high allele frequencies called by the GATK [46] were used for polymorphic marker. HRs were identified based on reciprocal change in mapped coverage and allele frequency, as described in Supplementary Fig. S2. To exclude false-positive recombinations, identical recombinations detected in the control strains and the other spores were eliminated. Since ∼95% of the NCOs in *SPO11^+^* spores were <5 kb in length, the COs and NCOs in MagTAQed spores were classified with the threshold of 5 kb in interval size. Consequently, 285 COs and 121 NCOs were detected in 32 MagTAQed spores dissected from 22 tetrads (Supplementary Table S6). Aneuploidies and CNVs were detected by mapping coverage and allele frequency. CNV regions were further analyzed by Nanopore long-read sequencing and *de novo* assembly (see below).

### Nanopore long-read sequencing and *de novo* assembly

High-molecular-weight DNA was extracted using Dr GenTLE™ High Recovery for Yeast Kit (Takara Bio Inc., Japan) and SRE XL KIT (Pacific Biosciences of California, Inc., USA), according to the manufacturer’s instructions. The library preparation was performed using Ligation Sequencing Kit (SQK-LSK109, Oxford Nanopore Technologies, UK) and NEBNext Companion Module for Oxford Nanopore Technologies Ligation Sequencing (E7180S, New England Biolabs, USA). The time series signal data in FAST5 format were acquired by Flow Cell R9.4.1 (FLO-MIN106D, Oxford Nanopore Technologies, UK) and Minknow software for MinION MkB (Oxford Nanopore Technologies, UK). FAST5 data were translated into sequence read data in FASTQ format using Guppy basecaller [50]. Short-read sequence data for improving the quality of genome assembly were acquired by Illumina HiSeqX Ten (Illumina, Inc., USA). The long-read sequence reads (> 30 kb, total 1 668 916 338 bp, N50 = 53 140 bp) were assembled into contigs by Canu version 2.2 [51]. The contigs were manually aligned by BLAST sequence search and dot plot by D-GENIES [52]. For correcting small assembly errors, we polished the genome assembly three times with Illumina short-read sequence reads by Pilon ver1.23 [53]. Tandem DUPs were detected by comparing to the S288C reference genome (sacCer3) in a dot plot by D-GENIES.

### Comparison of COs with chromatin features

Nucleosome-depleted regions (NDRs) were identified by deep sequencing data of micrococcal nuclease (MNase)-resistant mononucleosomes during meiosis [6]. First, the sequence reads were mapped to the SK1 genome using bowtie2 [54], followed by removing PCR duplicates using the Picard MarkDuplicates. Based on the genome-wide maps of nucleosomes, the NDRs were defined as the regions where no reads were mapped. The meiotic DSB hot spots were defined as NDRs that overlapped with Spo11-oligo peaks determined by the previous Spo11-oligo DNA sequencing, while cold spots were defined as NDRs without any mapped Spo11-oligo reads [6]. Spo11-oligo peaks were called by MACS2 (options: -g 1.2e7, -q 0.05, -nomodel, -extsize 147) [55]. To increase the resolution of CO positions, only CO events within 1 kb distance between markers were used in the analysis. Distances from the centers of all NDRs, hot spots, or cold spots to the endpoints of CO events were measured. Chromosome axes were identified by the previous genome-wide chromatin immunoprecipitation sequencing of meiotic cohesin Rec8 [28]. Rec8-enriched peaks were called using MACS2 (options: -g 1.2e7, -q 0.05, -nomodel, -extsize 147). The distance from the center of the Rec8-enriched peaks to the endpoints of CO events was measured. All *P*-values were calculated using the Mann–Whitney U test. All *P*-values were calculated using the Mann–Whitney U test.

## Results

### Development of photoactivatable MboI restriction enzyme MagMboI

**Figure 1.**
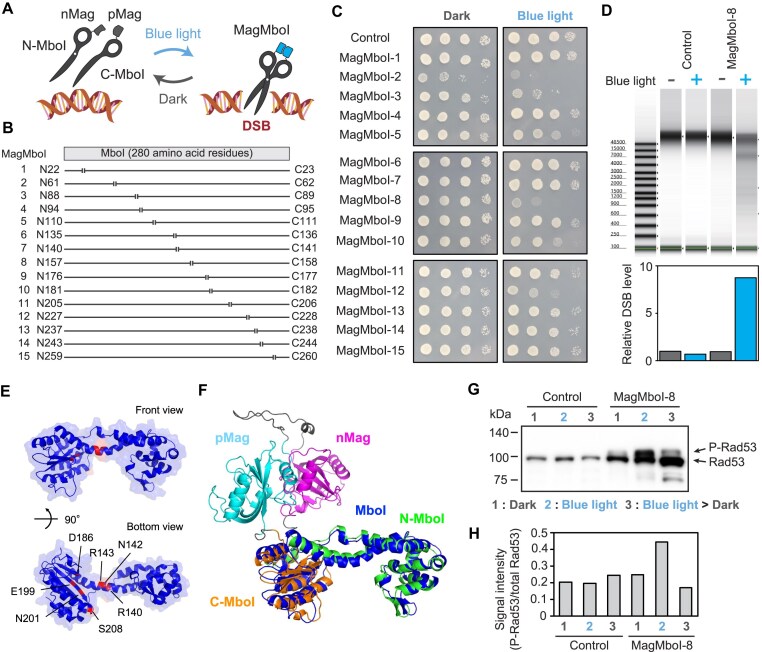
Development of photoactivatable endonuclease MagMboI. (**A**) Schematic diagram of photoactivatable endonuclease MagMboI. Restriction enzyme MboI was split into N-terminal (N-MboI) and C-terminal (C-MboI) fragments fused with paired photoswitchable proteins nMag (negative Mag) and pMag (positive Mag), respectively. N-MboI and C-MboI were disassociated and nonfunctional in the dark, whereas they form functional heterodimers (MagMboI) that induce DNA DSB upon light irradiation. (**B**) MagMboI variants with different split sites of MboI protein. (**C**) Viability assay examining the functional endonuclease activities of individual MagMboI variants depending on light irradiation. Ten-fold serial dilutions of the S288C strain with plasmid (Control) or MagMboI variants were spotted and cultured for 48 h at 30°C in the dark or upon light irradiation. (**D**) Electrophoresis assay for quantifying DSB level using the TapeStation system (Agilent Technologies). Relative DSB levels are normalized against the control (empty vector) without light exposure. (**E**) Two views of predicted structure of restriction enzyme MboI. The amino acid residues important for high DNA cleavage activity were shown in the figure (R140, N142, R143, D186, E199, N201, and S208) [61]. (**F**) Predicted structure of MagMboI-8 compared with that of MboI (blue). (**G**) Immunodetection of phosphorylated Rad53 (P-Rad53) with a C-terminal 3 × FLAG tag. (1) under dark conditions; (2) exposed to blue light for 30 min; (3) under dark conditions for 12 hours following light exposure. An uncropped image is in Supplementary Fig. S5. (**H**) Ratio of signal intensity of P-Rad53 to total Rad53.

To construct a photoactivatable restriction enzyme, we split a restriction enzyme MboI into two and linked each part with a set of light-induced dimerization module, “Magnets” (Fig. 1A). Magnets are composed of a couple of modified light-switch protein tags (pMag and nMag) [56–60], which are derived from a blue-light receptor VIVID of *Neurospora crassa*. These tags rapidly dimerize under exposure to blue light (wavelength: 470 nm) and revert to monomers when shielded from light. MboI is a restriction enzyme that recognizes and cleaves a specific four-base sequence (5′-GATC-3′). It has been demonstrated to induce a high DSB response *in vivo* compared to other restriction enzymes [9].

To identify the optimal split site of MboI, we prepared 15 types of vectors expressing fragments of MboI, each split at a different location and linked to Magnets (Fig. 1B). The vectors express the N-terminal fragment fused to nMag (N-MboI) and the C-terminal fragment linked with pMag (C-MboI), separated by an in-frame 2A self-cleaving peptide (P2A) (Supplementary Fig. S3).

To select the optimal MagMboI set that shows the most efficient light-dependent activity, we introduced each expression plasmid into a haploid *S. cerevisiae* strain to test the growth defect under blue light exposure for 48 h (Fig. 1C). Most of the MagMboI sets (except for MagMboI-2 and MagMboI-3) showed equivalent growth to the control (empty vector) under dark conditions due to the inactivation of DNA cleavage activity. Control cells with the empty vector exhibited the same growth rate under blue light as under dark conditions, indicating that the blue light exposure had little effect on yeast growth. MagMboI-2 and MagMboI-3 sets showed obvious growth defect under both dark and light conditions. In contrast, MagMboI-8 (splitting at 157th/158th amino acids) and MagMboI-12 (splitting at 227th/228th amino acids) sets showed equivalent growth rate to the control in dark conditions, but significant growth defects were observed only under blue light exposure. To compare the DSB levels among different constructs, we quantified the cleaved DNA extracted from each strain carrying each MagMboI construct using TapeStation system (Agilent Technologies). As a result, MagMboI-8 was the well-controlled variant that exhibited activity exclusively under blue light exposure (Fig. 1D). The results of other variants were shown in Supplementary Fig. S4. Since the 3D structure of MboI has not been elucidated, we predicted structural model of MboI using AlphaFold2 [44]. Mapping of amino acid residues important for DNA cleavage activity showed that the active site is located inside the structural groove of MboI [61] (Fig. 1E). Overlaying the structural model of MboI with that of MagMboI revealed that the dimerized pMag and nMag were on the opposite side of the active site (Fig. 1F). Therefore, it is likely that the split sets of MagMboI-8 can efficiently assemble to active enzymes only when exposed to blue light.

To verify whether MagMboI induces DNA damage response, phosphorylation of a DNA damage checkpoint protein Rad53 was examined by the band mobility shift in sodium dodecyl sulfate–polyacrylamide gel electrophoresis. While the band size of Rad53 in the control strain carrying the empty vector did not change upon blue light irradiation, the strain expressing MagMboI-8 showed a partial band shift (representing phosphorylated Rad53) in response to blue light irradiation, which diminished after returning to dark conditions (Fig. 1G and H, and Supplementary Fig. S5). These results indicate that MagMboI activates a DNA damage response upon blue light irradiation and is reversibly inactivated when returned to dark conditions.

### Chromosomal rearrangements induced by MagMboI in mitotic cells

**Figure 2.**
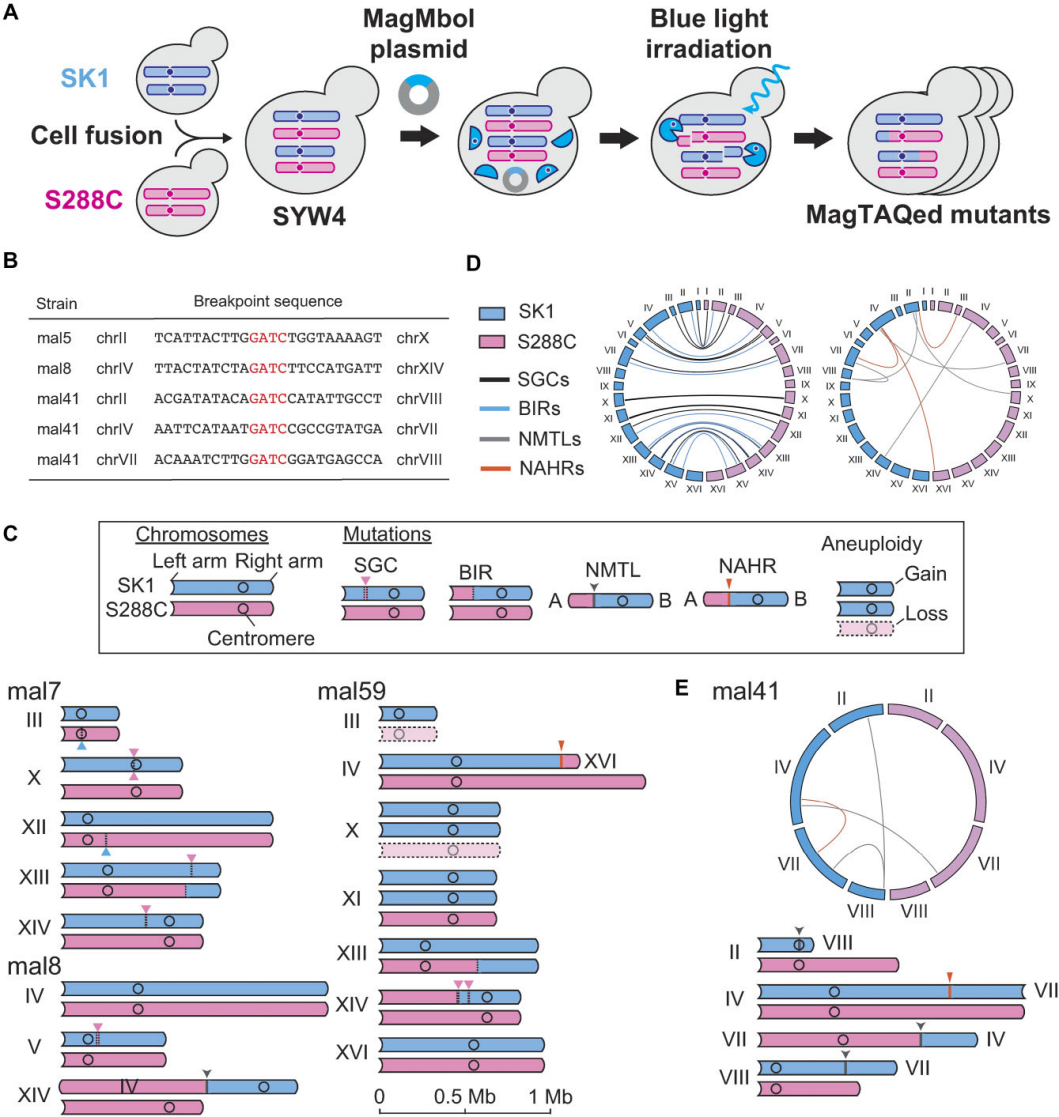
Diverse chromosomal rearrangements of MagTAQed mutants in mitosis. (**A**) A schematic diagram of MagTAQing system. A diploid SYW4 strain was constructed by cell fusion of the haploid strains SK1 and S288C. The SYW4 cells harboring a plasmid that expresses MagMboI-8 were exposed to light in the exponential growth phase for 30 min, followed by culturing on agar plates to obtain MagMboI-activated (MagTAQed) isolates from single colonies. (**B**) Breakpoint sequences of NMTLs in MagTAQed mutants. (**C**) Schematic diagrams of rearranged chromosomes in representative MagTAQed isolates. (**D**) Circos plots of short gene conversions (SGCs), BIRs, NMTLs, and NAHRs within MagTAQed isolates. (**E**) Complex chromosomal rearrangements in mal41.

We next introduced MagMboI-8 into a mitotic diploid yeast to verify its ability to induce genome rearrangements (Fig. 2A). For a detailed genome-wide identification of recombination sites, we used a diploid strain (SYW4), constructed by cell fusion of SK1-background and S288C-background haploid strains (both have the same mating type *MATa* to prevent spontaneous sporulation), each possessing about 0.7% single nucleotide polymorphisms [62]. The vector containing MagMboI-8 was introduced into SYW4, and after transcriptional activation for 4 h, blue light was irradiated for 30 min. After light irradiation, MagMboI-treated (MagTAQed) cells were grown on nutrient-rich plates for single-colony isolation. Of >1000 single colonies, 19 colonies showing altered cell morphology were selected for whole-genome analysis using the Illumina HiSeq X (>50 coverages; Illumina, USA) (Supplementary Fig. S6A and B).

As a result, from the analysis of 19 MagTAQed isolates, we detected 4 SNVs, 24 SGCs, 11 BIRs, 8 TLs, and 37 aneuploidies (Fig. 2B–D and Supplementary Fig. S7). TLs have been identified to occur through two distinct mechanisms: NHEJ-mediated TLs (NMTLs, 5 cases/19 isolates) and NAHRs (3 cases/19 isolates) between repetitive sequences such as Ty retrotransposons.

The MboI-recognition sequences (5′-GATC-3′) were present in all the NMTL breakpoints, with no DELs or insertions (Fig. 2B). This suggests that the MboI-recognition sequences cleaved by photoactivated MagMboI were directly ligated. The other type of TL is NAHR, mediated by HR involving repetitive sequences (Supplementary Fig. S8). The differences between NMTL and NAHR are described in Supplementary Fig. S1. As we reported previously, we detected NAHRs within Ty elements interspersed throughout the genome, confirming that repetitive sequences are a source of genomic instability. In particular, complex genome structure was observed due to multiple TLs in mal41 (Fig. 2E). In mal41, we estimated that at least seven DSBs were induced across five chromosomes, generating four rearranged chromosomes. All rearranged chromosomes had a single centromere, and no simultaneous DELs of homozygous alleles were detected. The presence of a single centromere per chromosome is explained by the lethal effects of malfunction of centromeres such as dicentric chromosome formation or centromere DEL. These findings indicates that light-controlled MagMboI-induced DSBs can cause complex chromosomal rearrangements. Furthermore, the activity of MagMboI, which induces genome-wide simultaneous DSBs and triggers recombination, can also be inferred from viability. It has been confirmed that viability decreases upon transient blue light irradiation compared to the control (Supplementary Fig. S9). Intriguingly, mal61 had a haploid set of 16 chromosomes with only seven small gene conversions but with no large rearrangements like COs (Supplementary Fig. S10A). The origin of the lost chromosomes differed for each chromosome, and the FACS analysis revealed that this strain had a haploid DNA composition (Supplementary Fig. S10B).

### Expression of MagMboI in meiotic cells

The original TAQing system requires the heat treatment to activate the restriction enzyme TaqI [8]. However, this treatment may lead to invasive effects on cell physiology such as meiotic progression. MagMboI can induce conditional DSBs in specific cells simply by exposing cells to blue light with minimal invasive treatments. It is known that meiotic *S. cerevisiae* cells have a higher frequency of HR than mitotic cells, which results in COs and NCOs, while illegitimate (nonhomologous) recombination such as TL is suppressed [63]. Hence, we introduced MagMboI-8 into meiotic cells to examine whether the spectrum of chromosomal rearrangements was influenced by the types of cell division. To exclude the influence of authentic meiotic recombination, MagMboI-8 was introduced into diploid yeast lacking the *SPO11* gene, which is essential for the meiotic DSB formation and initiation of meiotic recombination. Our previous research demonstrated that the *spo11*Δ strain frequently produces aneuploid spores without any meiotic recombinations [42].

**Figure 3.**
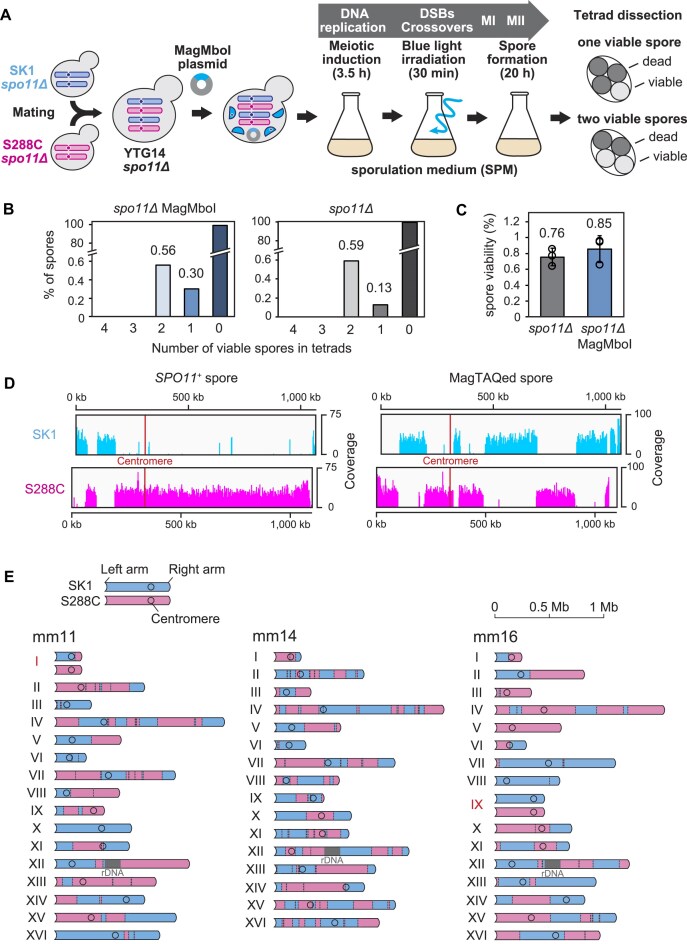
Meiotic recombination induced by MagMboI. (**A**) Induction of Spo11-independent meiotic DSBs with MagMboI. The MagMboI8-expression vector was introduced into the hybrid strain (YTG14 *spo11Δ*) constructed by crossing of the SK1 and S288C lacking *SPO11*. The hybrid cells induced to enter meiosis for 3.5 h, which corresponds to the timing of meiotic DSB, were irradiated with light for 30 min. After light irradiation, cells were cultured in the dark for 20 h to complete meiotic division (Meiosis-I, MI; Meiosis-II, MII) and spore formation. Spore viability was measured by tetrad dissection. (**B**) Viable spore numbers in dissected tetrad of *spo11Δ* (*n*= 12 619) and *spo11Δ* MagMboI (*n*= 3 948). Data of *SPO11^+^* spores are from Kawashima *et al.* (2023) [42]. (**C**) Spore viability of *spo11Δ* (0.76%) and *spo11Δ* MagMboI (0.85%) tetrads. Error bars represent standard deviations (*n*= 3). (**D**) Coverage plots of recombined chromosome XV in a WT *SPO11^+^* spore (wn1d) and a MagTAQed spore (mm14). The other three WT spores from the same tetrad is in the Supplementary Fig. S13. (**E**) Schematic diagrams of recombined chromosomes in MagTAQed spores. The names of aneuploid chromosomes are highlighted (mm11: chr I, mm16:chr IX).

To identify recombination sites during meiosis, we used a hybrid diploid *spo11*Δ strain (YTG14 *spo11*Δ), previously constructed by mating of SK1 and S288C-background haploids (Fig. 3A) [42]. This strain was transferred to SPM and after 3.5 h at the expected timing of Spo11-DSB formation, we exposed it to blue light for 30 min. The timing of blue-light exposure corresponded to the peak of DSB formation reported in previous studies [64]. Combining this finding with our data on meiotic progression in hybrid strains (Supplementary Fig. S11), we finalized the timing of blue-light exposure. When meiotic cells expressing MagMboI-8 were exposed to blue light, there was little effect on changes in DNA content compared to the mock control without blue light exposure, but there was a slight delay in the accumulation of cells that completed meiosis I and meiosis II within the range of 6–10 h (Supplementary Fig. S11). This delay is thought to reflect the DSBs induced by photoactivatable MagMboI.

After the blue light exposure, cells were further cultured in SPM for 20 h to complete meiosis, followed by tetrad dissection and whole-genome sequencing of viable spores (Fig. 3A). MagMboI-activated *spo11Δ* hybrids produced many tetrads without viable spores and few one or two viable spore tetrads. (total spore viability was 0.85%) (Fig. 3B and C). The spore viability of the *spo11Δ* hybrid reported by Kawashima *et al.* was 0.76% [42], suggesting that meiotic DSB induced by photoactivated MagMboI does not rescue spore viability in Spo11-less meiosis (*P* = .45, two-tailed Student’s *t*-test, *n*= 3) (Fig. 3B and C). To compare the spore viability under the same conditions related to the blue light, random spore analysis was performed. Both “*spo11Δ*” and “s*po11Δ* MagMboI" exhibited very low viability (<1%) irrespective of blue right irradiation (Supplementary Fig. S12).

### Meiotic recombinations in viable MagTAQed spores

Analyzing the whole-genome sequences of the rare viable spores, we detected multiple recombination events estimated as COs and NCOs per chromosome (Fig. 3D and E and Supplementary Figs S13 and S14). The rare viable spores in which recombination occurred represent only one of the four spores, as the corresponding homologous spore is not viable. Therefore, conventional tetrad analysis for detecting COs is not feasible in this study, and recombination events were identified solely based on SNP markers. As a result, reciprocal COs could not be detected, highlighting a limitation of our study. For example, when estimating the origins of chromosome XV in the MagTAQed spore mm14, we observed that chromosome sequences derived from the SK1 and S288C strains had undergone at least nine sequence exchanges estimated as COs (Fig. 3D). The sequence length of such chromosomal exchanges ranges from 20 kb to several hundred kb, much longer than a typical NCO (i.e. SGC).

**Figure 4.**
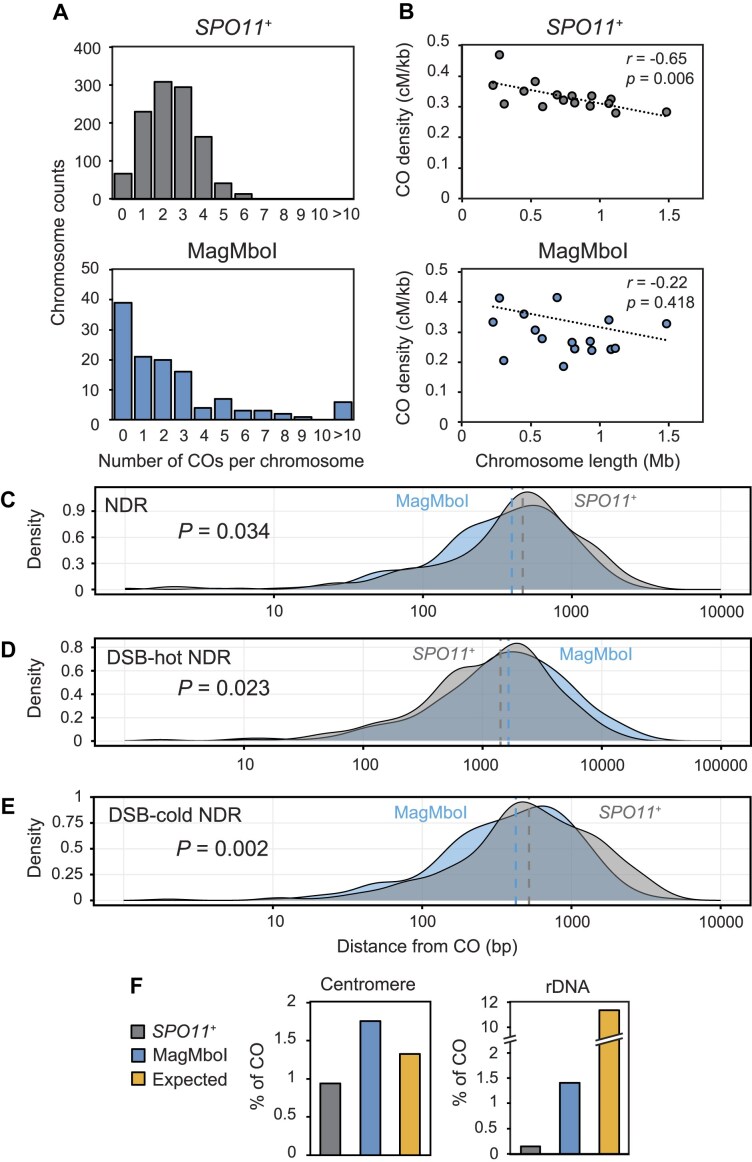
Distinct pattern of MagMboI-induced meiotic recombination. (**A**) The distribution of detected CO counts per chromosome in *SPO11^+^* and MagTAQed spores. (**B**) Correlation (Pearson’s *r*) between CO density (centimorgans per kb) and chromosome length (Mb). (**C–E**) Distance from *SPO11^+^* (*n*= 254) and MagMboI (*n*= 236) COs to (**C**) NDR, (**D**) Spo11-induced DSB hot spot (DSB-hot NDR), and (**E**) cold spot (DSB-cold NDR). *P* values were calculated using a Mann–Whitney U test. Dotted lines indicate median. Data of *SPO11^+^* spores was based on Kawashima *et al.* (2023) [42].–(**F**) Percentage of COs within centromere-proximal (±5 kb) and rDNA regions. Expected values indicate the percentage of the length of each chromosomal region (Centromere, 160 kb; rDNA, 1365 kb) in the total genome (12 Mb).

In normal meiosis of *S. cerevisiae*, the frequency of CO is controlled to occur between one to a few times on a single chromosome regardless of chromosome length [3, 65, 66]. Most of the CO counts per chromosome were 1–3 times in *SPO11*^+^ spores, whereas estimated number of MagMboI-induced CO (MagMboI-CO) varied widely from 0–20 times (Fig. 4A). In this study, the CO count represents the number of switches rather than the number of segments. CO density (cM/kb) therefore correlated negatively with chromosome length (*r* = −0.65, *P*= .006) in *SPO11*^+^ spores, whereas there was no statistically significant correlation between CO density and chromosome length (*P*= .418) in the MagTAQed spores (Fig. 4B). Notably, a few chromosomes (e.g. chromosome X in mm11) harbored no detectable COs, indicating no assurance of obligatory CO formation (Fig. 3E). In summary, MagMboI-CO is not as tightly regulated as Spo11-induced CO (Spo11-CO), suggesting that the number and distribution of CO are more random.

The ratio of one viable spore increased slightly compared to that of the control *spo11Δ* (0.13% versus 0.30%) but remained at a very low frequency. Therefore, in *S. cerevisiae*, MagMboI-induced meiotic DSBs can induce recombination, but cannot sufficiently rescue the spore viability, unlike nematodes and fission yeast [35–37]. In nematodes and fission yeast, which possess fewer chromosomes, chromosome pairing is achieved through recombination-independent systems. Additionally, the three studies involving *spo11Δ* strains employed distinct procedures for inducing DSBs: Dernburg *et al.* used radiation for *Caenorhabditis elegans* [35], Farah *et al.* utilized flap endonuclease mutants as a source of endogenous DSBs [36], while Pauklin *et al.* induced DSBs using exogenous activation-induced deaminase in nematodes and fission yeast [37]. No meiotic recombination was detected in the two viable spore tetrads, both of whose spore often showed identical patterns of aneuploidies (Supplementary Fig. S15A and B). The previous study by Kawashima *et al.* [42] reported the same feature in the sporulation of *spo11Δ* caused by a random assortment of homologous chromosomes during meiosis I and equal segregation of meiosis II. We assume that these identical ploidy sets of two viable spores were presumably due to a lack of MagMboI-8 activation with insufficient exposure to blue light during the sporulation culture.

Next, we examined the relationship between recombination sites and the higher-order structure of chromosomes. Meiotic chromosomes form a higher-order structure known as the axis-loop. The axis is composed of the cohesin complex that adheres to sister chromatids and other axis proteins interacting with Spo11 core complex [6, 24, 26–28, 67–70]. On the other hand, since DSB hotspots exist in the loop regions, it is thought that the axis and loop are at least in part physically connected during DSB formation [22, 23]. When we examined the distance between the MagMboI-CO and the Rec8 binding sites representing the axis, no statistically significant difference was observed in comparison with the distribution of recombination sites in *SPO11*^+^ (Supplementary Fig. S16).

Spo11-induced DSB hotspots are preferentially localized in NDRs [6, 29, 30], since Spo11 and Spo11-associated DSB proteins can easily access DNA. However, not all NDRs are Spo11 hotspots, and some NDRs are hardly subject to Spo11-induced DSBs. We examined the distance between these NDRs and the recombination sites. The distance between all NDRs and MagMboI-COs was not significantly different from that of Spo11-COs. This suggests that MagMboI induces DSBs in NDRs similarly to Spo11 (Fig. 4C).

The NDRs with or without Spo11-induced DSBs were identified by Spo11-bound oligonucleotide (Spo11-oligo) sequencing [6]. We thus compared the distances between MagMboI-COs and DSB-hot NDRs (with Spo11-oligo reads) or DSB-cold NDRs (without Spo11-oligo reads). The results showed that the distance of MagMboI-COs to DSB-cold NDRs was statistically shorter than that to Spo11-COs (Fig. 4D and E). These results suggest that MagMboI can introduces DSBs in DSB-cold NDRs as well as in DSB-hot NDRs.

To further confirm this feature, we examined the frequency of MagMboI-COs in specific chromosomal regions where meiotic recombination is suppressed. In this study, COs involving the rDNA region were quantified based on a single event when a shift in chromosome origin was identified at the boundary of the rDNA region. As a result, consecutive COs occurring through the rDNA region, if present, remain undetectable under this approach. The centromere-proximal (±10 kb) regions and rDNA are chromosomal regions where Spo11-induced DSBs are hardly detected [6]. The proportions of these regions in the genome are 1.3% and 11.3%, respectively (indicated by yellow bars in Fig. 4F), and these proportions are the expected CO frequencies if COs occurred uniformly across all chromosomes. Spo11-COs (gray bars) are about 0.9% in the centromere-proximal region and about 0.1% in rDNA, which is lower than the expected frequencies, indicating that recombination is suppressed (Fig. 4F). On the other hand, the frequency of MagMboI-COs is 1.8% at the centromere-proximal region, which is comparable to the expected frequency. In the rDNA region, the frequency of MagMboI-COs is 1.4%, about eight-fold lower than the expected frequency, but still 14-fold higher than the frequency of Spo11-COs (Fig. 4F). These results suggest that MagMboI-induced DSBs are much less influenced by recombination suppression in centromere-proximal and rDNA regions.

### Noncanonical recombinations between repetitive sequences

**Figure 5.**
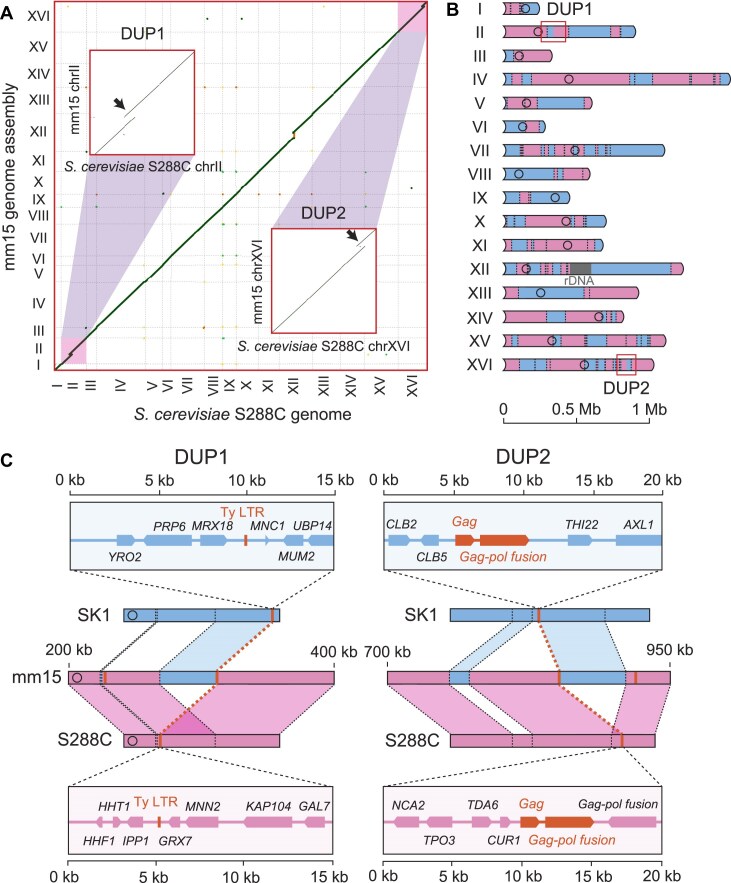
Aberrant meiotic recombination in MagTAQed spores. (**A**) Comparison of the *S. cerevisiae* S288C genome and mm15 genome assembly. Magnified views of comparison for chromosome II and XVI show tandem DUPs (DUP1 and DUP2) indicated by arrows. (**B**) Schematic diagram of recombined chromosomes in mm15. DUP1 and DUP2 regions are shown in the boxes. (**C**) Magnified views of DUP1 and DUP2. Both tandem DUPs were caused by HRs between homologues and NAHRs between repetitive sequences.

Interestingly, partial increases in sequence coverage were observed in chromosomes II and XVI of mm15, suggesting that these chromosomal regions were duplicated by noncanonical recombination (Supplementary Fig. S15A, blue arrowheads). Therefore, we used long-read Nanopore sequencing to construct *de novo* genome assembly of mm15. Comparative analysis with the reference genome showed tandem DUPs in chromosomes II and XVI (Fig. 5A and B, named DUP1 and DUP2, respectively). Importantly, both DUP1 and DUP2 were due to NAHR between repetitive sequences associated with Ty retrotransposon (Fig. 5C). These findings revealed that meiotic DSB induced by MagMboI results in not only allelic HR but also recombination between nonallelic homologous sequences, thereby causing chromosomal abnormalities more frequently.

### Chromosomal aberrations in return-to-growth isolates

**Figure 6.**
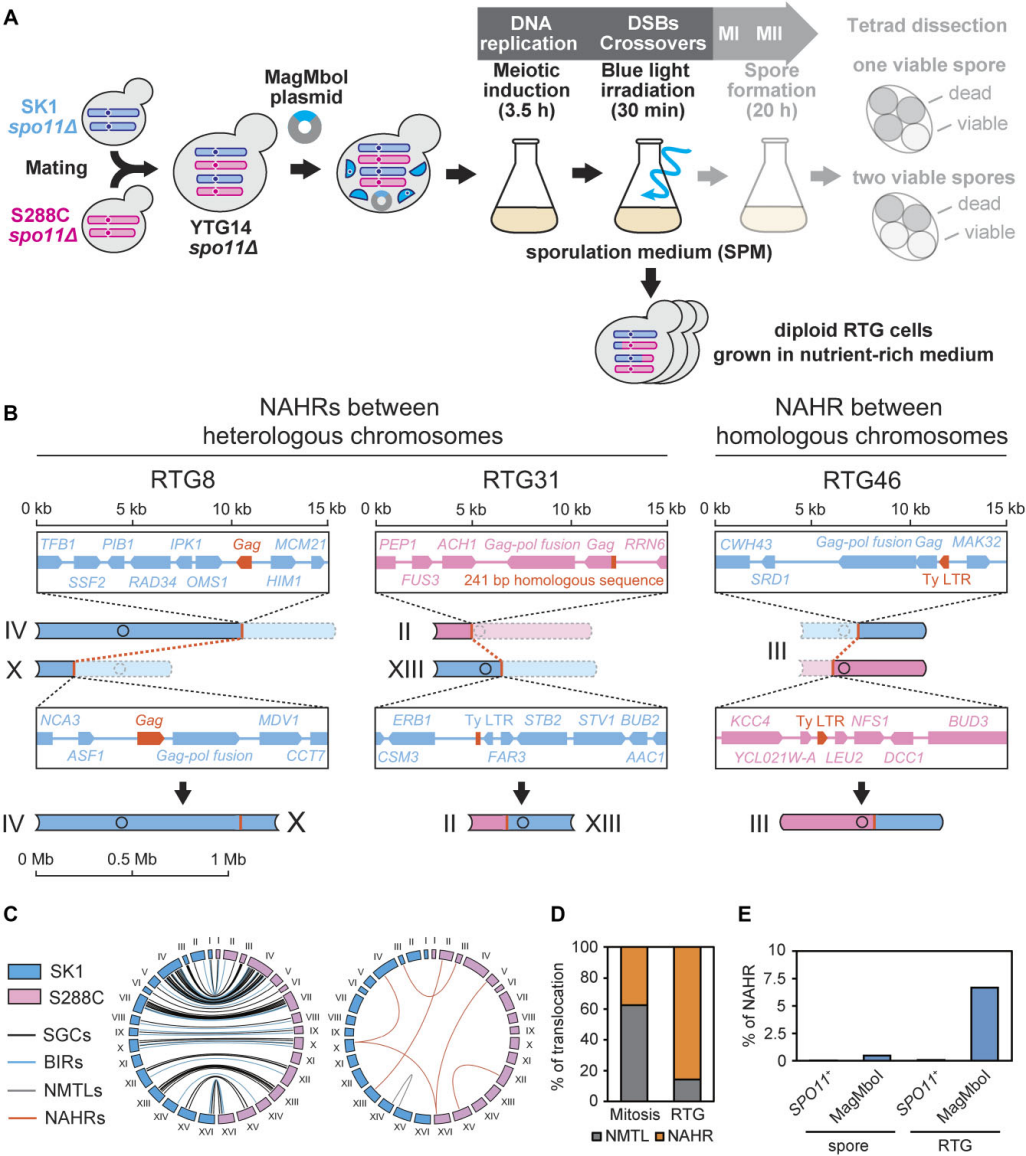
MagMboI-induced recombination cause chromosomal abnormalities. (**A**) A schematic diagram of RTG experiment to isolate the diploid strains with rearranged chromosomes induced by MagMboI. (**B**) NAHRs between heterologous chromosomes in RTG8 and 31. NAHR between homologous chromosomes in RTG46. (**C**) Circos plots of SGCs, BIRs, NMTLs, and NAHRs within MagTAQed RTG isolates. (**D**) Increased percentage of NAHR per HR in RTG isolates compared to mitotic isolates. (**E**) Percentage of detected NAHRs among recombination events. Data of NAHR in *SPO11^+^* RTG cells were reported by Laureau *et al.* (2016) [73].

MagMboI-induced meiotic NAHRs led us to hypothesize that lethal chromosomal abnormalities decreased spore viability. We considered that analyzing the genome sequences of viable spores could not detect these fatal chromosomal rearrangements. Therefore, a return-to-growth (RTG) experiment [71], a recovery of meiotic cells from sporulation culture to the vegetative mitotic cell cycle in a nutrient-rich condition, was employed to analyze the formation of chromosomal abnormalities during meiosis I. In RTG experiments, meiotic cells exposed to blue light were immediately transferred to nutrient-rich plates to isolate MagTAQed RTG cells (Fig. 6A). The genome analysis revealed MagTAQed RTG isolates underwent multiple LOHs due to HR (Supplementary Fig. S17 and Supplementary Table S4). Since recombination does not occur in *spo11Δ* [42, 72], we conclude that MagMboI indeed induced recombination in the RTG experiment.

Laureau *et al.* showed that numerous NCOs and a few COs per chromosome were observed in *SPO11^+^* RTG strains [73]. The blue-light-exposed *spo11*Δ diploids expressing MagMboI-8 also generated multiple NCOs. We also detected BIR events possibly reflecting COs but at a lower frequency than in *SPO11*^+^ diploid (Supplementary Fig. S17). Note that some chromosomes had no NCOs or COs detected, suggesting that MagMboI-8 randomly induces DSBs.

Notably, most TLs (six out of seven) were due to NAHR between Ty retrotransposons, with the remaining case being an NMTL at the MboI-recognition sequence (5′-GATC-3′) (Fig. 6B and C  Supplementary Fig. S18). The frequency of NAHR was higher in MagTAQed RTG isolates than in mitotic MagTAQed isolates, likely reflecting elevated homology-directed repair pathway in meiotic cells (Fig. 6D). More importantly, NAHRs were detected in the MagTAQed spores (0.49%) and MagTAQed RTG isolates (6.67%) at a much higher frequency than *SPO11*^+^ spores (0.03%) and *SPO11^+^* RTG cells (0.08%), respectively (Fig. 6E). These results suggest that NAHRs are presumed to induce nondisjunction of chromosomes and genomic instability during sporulation, possibly leading to a decrease in spore viability.

## Discussion

### Development of optogenetic system conditionally inducing large-scale genome rearrangements

We developed a photoactivatable endonuclease MagMboI, which allows conditional induction of large-scale genome rearrangements. Regarding the kinetics of photoactivation, it was confirmed that DNA cleavage ability of MagMboI can be activated within 30 min after light exposure and loses activity within 12 h after the light shielding (Fig. 1G and H). Although the deactivation under dark conditions is relatively slow, using recently reported pMagFast1 and nMagFast1, which have a faster dissociation rate (*t*_1/2_ = 4.2 min), could enable more responsive induction of genome rearrangement in the future [56].

There are several technologies related to MagTAQing, such as SCRaMbLE (Synthetic Chromosome Rearrangement and Modification by LoxP-mediated Evolution) [74] and multi-target CRISPR/Cas9 [75–77]. However, SCRaMbLE requires time-consuming synthesis steps of cells harboring artificial chromosomes with insertions of multiple LoxP sequences. Multi-target CRISPR/Cas9 reportedly induces structural variants such as TLs by targeting transposons, centromeres, and other repetitive sequences. However, it is challenging to induce random rearrangements across all chromosomal regions. In contrast, MagTAQing can be easily implemented in any species capable of incorporating MagMboI, offering high versatility. In addition, since MagTAQing uses four-base recognition restriction enzyme, it can induce genome rearrangements at more diverse sites, facilitating the acquisition of mutant strains with altered phenotypes.

The MagTAQing method enables genome rearrangements in specific cells by temporally and spatially controlled blue light irradiation, revealing spectra of genome rearrangements in specific differentiated cells or dependent on cell cycle phases. Human cancer cells tend to have more complex chromosome rearrangements than normal diploid cells, often with tissue-specific rearrangement patterns such as *BCR*-*ABL* or *MLL* rearrangements [78]. In the future we can locally activate MagTAQing *in vivo* by targeted blue light irradiation with optical fibers, potentially enabling the investigation of cancer-specific rearrangements and chromothripsis.

### Comparison of genome rearrangements induced by MagMboI during mitosis and meiosis

Using MagTAQing, we compared genome rearrangement patterns during mitosis and meiosis. Environmental factors such as temperature changes and media exchange are expected to influence meiotic progression in yeast. For instance, increased temperatures affect meiotic recombination [79, 80]. Therefore, the original TAQing system, which activates the restriction enzyme TaqI by an increase in temperature, could not be applied to meiosis. In contrast, activation of MagMboI requires only blue light exposure, which has very low invasiveness to meiotic yeast cells. We introduced MagMboI into Spo11-deficient meiotic cells to examine differences in DNA repair and genome rearrangements between mitosis and meiosis. MagTAQed meiotic cells underwent a higher frequency of NAHRs leading to interchromosomal TLs and tandem DUPs in homologous regions than mitotic cells (Figs 5 and 6B–E). This finding suggests that meiosis suppresses NHEJ and increases HR frequency, attributed to the activation of HR enzymes like Rad51 and Dmc1 and the proximity of homologous chromosomes during meiosis [38].

An intriguing case of MagTAQing-induced genome rearrangements during mitosis is haploidization (Supplementary Fig. S10). The diploid strain used in the mitotic MagTAQing experiments has a *MATa/a* mating type, ruling out the possibility of haploidization through meiosis. Additionally, if meiosis had occurred, HRs should be detectable in most chromosomes; however, HRs were rare and small-scale in mal61. A similar phenomenon has been observed during serial cultivation of *rad52Δ* diploid [81], where unrepaired DSB ends accumulate leading to haploidization. Although the diploid strain used in this study has the wild-type *RAD52*, it is possible that the extensive DSBs generated by MagTAQing were not fully repaired, leading to haploidization. This phenomenon significantly elevated the total count of aneuploidies among the 19 MagTAQed isolates, as we counted the haploidized 16 chromosomes in mal61 as 16 aneuploidies. Aneuploidy formation has also been proposed to occur at a relatively high frequency, since the presence of extra chromosomes in aneuploid cells would confer genetic redundancy and advantages such as increased stress tolerance, beyond morphological traits [82], potentially enhancing their survival likelihood. Furthermore, MagMboI is capable of inducing DSBs randomly, including in the proximal region of the centromere, which is hypothesized to contribute to the elevated frequency of aneuploidy [83].

### Differences in consequence of DSBs induced by Spo11 and MagMboI

Spo11-deficient meiotic cells exhibit abnormal gamete formation due to improper homologous chromosome segregation [42, 72, 84, 85]. However, DSB induction can partially restore meiosis in Spo11-deficient nematode and fission yeast [35–37]. While MagTAQing induced significant HR in Spo11-deficient cells, high frequencies of chromosomal abnormalities were observed, and spore viability remained low, suggesting that MagMboI-induced DSBs are insufficient to rescue normal meiosis in Spo11-deficient *S. cerevisiae* cells (Fig. 3B).

For this, two explanations are possible: first, MagTAQing may not induce recombination at levels necessary for proper homologous chromosome segregation; however, this may not be the case since numerous recombinations between homologous chromosomes were induced by MagMboI activation (Fig. 3). Second, there may be qualitative differences between recombination induced by MagMboI and Spo11, affecting segregation accuracy. We therefore examined various features of recombination induced by MagMboI and Spo11 to test this possibility.

**Figure 7.**
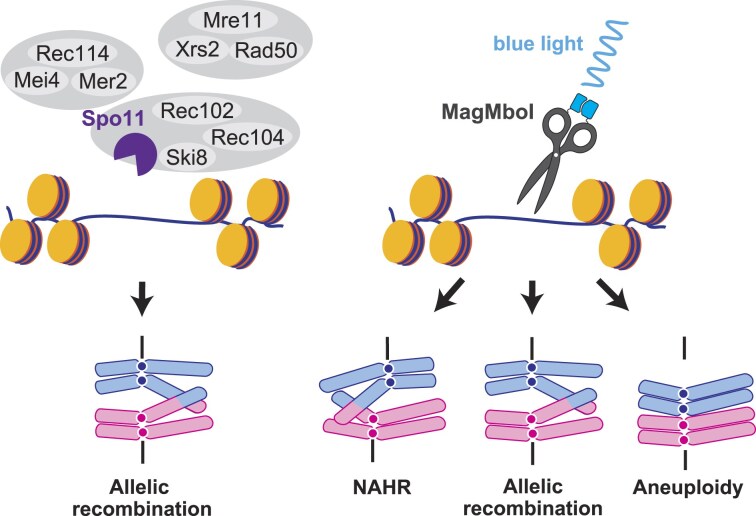
Model for Spo11 and MagMboI-induced meiotic recombination. (**Left**) A schematic diagram of Spo11 complex machinery. Spo11 binds to Ski8, Rec102, and Rec104 to form the core complex associated with the RMM proteins (Rec114, Mei4, and Mer2) and the MRX complex (Mre11, Rad50, and Xrs2) during DSB induction. (**Right**) A schematic diagram of MagMboI-induced chromosomal abnormality.

First, spatial control of DSBs and accompanying recombination sites was examined. Spo11 preferentially induces DSBs in NDR [6, 28–30]. Recombination sites by MagMboI and Spo11 showed similar correlations with NDR, suggesting MagMboI also induces DSBs in NDR (Fig. 4C). Spo11 forms DSBs in collaboration with several accessory factors. In budding yeast, a core complex with Ski8, Rec102, and Rec104 is formed, with DNA cleavage requiring MRX complex (Mre11, Rad50, and Xrs2) and RMM complex (Rec114, Mei4, and Mer2) interactions [86]. These factors, closely linked with histone modifications, chromosomal structural changes, and DNA replication cycles, suggest coordinated progression of meiosis, meiotic recombination, and spore formation [38, 76]. In contrast, MagMboI-induced DSBs are not under such complex regulation, potentially leading to a higher incidence of chromosomal abnormalities (Fig. 7). For instance, during DSB formation by Spo11, there are DSB-hot NDRs and DSB-cold NDRs, suggesting activity control at chromosomal domain levels. Spo11-oligos are exclusively found in DSB-hot NDRs, whereas MagMboI-CO sites were significantly farther from DSB-hot NDRs compared to Spo11-CO sites and are statistically closer to DSB-cold NDRs (Fig. 4D and E). This suggests that MagMboI can form DSBs in DSB-cold NDRs that Spo11 cannot function.

Additionally, DSB formation by Spo11 is controlled by meiotic chromosome-specific axis-loop structures. Spo11 accessory proteins are mostly located in the axis, while DSB hotspots are observed in the loop. During recombination initiation, the loop is assumed to interact with the axis, converting Spo11 to an active state, which introduces DSBs at hotspots in the loop. Rec8, a kleisin subunit of meiotic-specific cohesin contributes as a base for forming the axis-loop structure. The distribution of *S. cerevisiae* Spo11 is regulated in close association with Rec8 binding sites [24]. Moreover, when Spo11 fused with a sequence-specific DNA binding domain of Gal4 is targeted to the loop or axis regions, DSB activity is suppressed only in the axis regions, indicating that DSB formation in the axis is repressed [28]. We observed that the distance between MagMboI-CO sites and Rec8 binding sites was comparable to that of Spo11-CO sites (Supplementary Fig. S16), suggesting that the axis-loop structure may affect MagMboI-COs. Since the axis is also the site for sister chromatid cohesion, MagMboI-induced DSBs in the axis may initiate inter-sister recombination which is indistinguishable by genome sequencing.

Spo11-induced DSBs are recombined in specific modes. For example, CO homeostasis ensures a couple of COs per chromosome regardless of the strength of recombination initiating function of Spo11 [65], obligate CO guarantees at least one CO per chromosome [66], and CO interference prevents closely spaced COs [87–90]. The plots of CO frequency versus chromosome length demonstrated that Spo11 induces about 2–3 COs per chromosome independent of chromosome length, whereas MagMboI-induced DSBs follow a power-law distribution, suggesting the stochastic occurrence of COs without controls of CO homeostasis and assurance (Fig. 4A and B). Notably, some chromosomes showed no recombination with MagMboI leading to the generation of spores with aneuploidy, suggesting stochastic DSB formation alone is insufficient for stable chromosome segregation (Fig. 3D, mm11 chromosome X, mm16 chromosome IX). Furthermore, many closely spaced COs were observed in MagTAQed cells, suggesting that there is no CO interference (Fig. 3D). It is known that Spo11-induced COs include interference-dependent Type I and interference-independent Type II [90–92]. MagMboI-induced COs are likely to be predominantly Type II. Altogether, features of recombination by MagMboI-induced DSBs are different from those by Spo11-induced DSBs, thereby possibly causing chromosome aberrations during meiosis.

### Species-dependent response to artificial DSBs in spo11 deficient cells

In organisms such as nematodes and fission yeast, it has been reported that the introduction of artificial DSBs can partially rescue meiotic defects caused by Spo11 deficiency [35–37]. However, in *S. cerevisiae*, MagMboI-induced DSBs during meiosis did not significantly improve the spore viability. One explanation for such a difference is the uniqueness of meiosis in those species.

In nematodes, fruit flies, and fission yeast, specific mechanisms are known to facilitate homologous chromosome pairing during meiosis. For example, in nematodes, there are regions called “pairing centers” at the ends of chromosomes, where specific DNA-binding proteins bind to mediate homologous chromosome pairing [93]. In fission yeast, meiosis-specific noncoding RNAs are transcribed and function as “pairing centers” through binding proteins to hold homologous chromosomes together. Additionally, the “horsetail movement”, a back-and-forth nuclear movement, assists in homologous chromosome pairing and recombination [94, 95]. These organisms have mechanisms for homologous chromosome pairing that do not rely on DSB induction, making them more amenable to functional recovery through artificial DSBs.

Moreover, these organisms have fewer chromosomes compared to budding yeast (*n*= 16), with nematodes having six chromosomes and fission yeast having three chromosomes. In Spo11-mediated DSB formation, a nearly equal number of DSBs are induced on all chromosomes regardless of their length. In contrast, artificial DSBs occur stochastically, meaning that shorter chromosomes have a lower frequency of DSB formation, resulting in fewer or little COs (Fig. 4A and B). In species with many chromosomes and larger chromosome length variation, artificial DSBs might not consistently induce COs across all chromosomes.

### Spo11-independent recombination and chromosomal instability

MagMboI-induced DSBs during meiosis resulted in chromosomal aberrations which rarely occurred in cells with Spo11-induced DSBs (Figs 5 and 6B–E). MagMboI induced meiotic recombination in centromeres, rDNA, and retrotransposons. Recombination near centromeres, leading to abnormal chromosome segregation, is suppressed in Spo11-mediated meiotic recombination [6, 96]. Ctf19, a kinetochore component, inhibits Spo11-induced DSBs near centromeres [97]. In contrast, MagMboI could form DSBs near centromeres, inducing recombination at high frequencies (Fig. 4F), independent of Spo11-related factors, which can result in formation of nonviable spores due to chromosome mis-segregation. MagMboI also promoted recombination in rDNA (Fig. 4F). Histone deacetylase Sir2 is thought to suppress meiotic recombination in rDNA by excluding Hop1 from rDNA, which likely prevents Spo11-induced DSBs [98–101]. However, it is possible that MagMboI induces DSBs independently of Hop1, potentially enabling recombination in rDNA.

MagMboI-induced DSBs can frequently induce NAHR of Ty retrotransposons (Figs 5C and 6E). RTG experiments suggested that meiotic cells with NAHRs induced by MagMboI largely resulted in inviable spores, possibly due to the chromosomal missegregation (Fig. 6E). Such NAHRs forming lethal spores were suppressed in *SPO11^+^* normal meiosis [5]. Spo11-induced DSB formation is suppressed about 15-fold lower in Ty compared to the genome average and two- to three-fold lower than in open reading frames [6, 102]. Additionally, inserting Ty sequences near DSB hotspots such as *HIS4* suppresses Spo11-induced DSB formation and recombination [4]. The precise molecular mechanisms of recombination suppression by Ty are unclear, but the involvement of Sir2-mediated chromatin silencing, like that in rDNA, has been suggested [103]. Such regulation is irrelevant to MagMboI, which forms DSBs stochastically across the genome, leading to frequent NAHR-associated unequal COs.

Repetitive sequences such as retrotransposons constitute more than half of the human genome. NAHR via these repetitive sequences is a universal phenomenon, as evidenced by the detection of a few NAHR events per cell in somatic tissues of healthy individuals [104]. However, NAHR in both somatic and germline tissues can lead to various human diseases, including cancer and neurological disorders, making the elucidation of the NAHR mechanisms crucial for a systematic understanding of these diseases [5, 105, 106].

Compared to somatic cells, meiotic cells favor DNA repair through HR mechanisms. Therefore, the suppression of NAHR-induced chromosomal abnormalities is expected to be even more critical during meiosis than in mitosis (Fig. 6D). The increased frequency of NAHR resulting from Spo11-independent DSBs, as observed in this study, suggests that the stringent control of DSB formation by Spo11 and its accessory factors play a more pivotal role in maintaining genome integrity than previously thought.

## Supplementary Material

gkaf206_Supplemental_Files

## Data Availability

Sequencing data were deposited in the DDBJ/EMBL/GenBank database under BioProject accession number PRJDB17959. Assembled sequence data of YPH499 and S799 strains were deposited in the DDBJ/EMBL/GenBank database under BioProject accession number PRJDB5253. All other data are available from corresponding authors upon request.

## Notes

Present Address: Department of Cellular Biology, Research Institute for Radiation Biology and Medicine, Hiroshima University, Hiroshima 734-8553, Japan
