# Supplementary material for: Light-controlled Spo11-less meiotic DNA breaks by MagTAQing lead to chromosomal aberrations
Source: Nucleic Acids Res. 2025 Apr 10;53(7):gkaf206. doi: 10.1093/nar/gkaf206 (PMC11983132; doi:10.1093/nar/gkaf206)
Supplement: gkaf206_Supplemental_Files [file gkaf206_supplemental_files.zip › MagTAQing_NAR_supplementary_data_revised.pdf]

Supplementary data

Light-controlled Spo11-less meiotic DNA breaks by MagTAQing lead to chromosomal aberrations

Hideyuki Yone[1,*,†], Yuri Kawashima[1,†], Hayato Hirai[1], Arisa H. Oda[1], Moritoshi Sato[1,2], Hiromitsu Kono[1], and Kunihiro Ohta[1,3,*]

[1]Department of Life Sciences, Graduate School of Arts and Sciences, The University of Tokyo, Komaba 3-8-1, Meguro-ku, Tokyo 153-8902, Japan

[2]Kanagawa Institute of Industrial Science and Technology (KISTEC), 3-2-1 Sakado, Takatsu-ku, Kawasaki, Kanagawa 213-0012, Japan

[3]Universal Biology Institute, The University of Tokyo, Hongo 7-3-1, Bunkyo-Ku, Tokyo 113-0033, Japan

* To whom correspondence should be addressed. Tel & Fax: +86 22 24828724; Email: kohta-pub2@bio.c.u-tokyo.ac.jp

Correspondence may also be addressed to Hideyuki Yone. Email: hyone@g.ecc.u-tokyo.ac.jp

[†] The first two authors should be regarded as Joint First Authors.

Present Address: Yuri Kawashima, Department of Cellular Biology, Research Institute for Radiation Biology and Medicine, Hiroshima University, Hiroshima City, Japan

Description of supplementary data

Supplementary figures

Supplementary FigureS1. Bioinformatic workflow to characterize the mutation events in the MagTAQed diploid strains.

Supplementary Figure S2. Bioinformatic workflow to characterize the mutation events in the MagTAQed haploid spores.

Supplementary Figure S3. Constructs of MagMboI.

Supplementary Figure S4. Quantification of cleaved DNA for MagMboI variants.

Supplementary Figure S5. Uncropped image used in Figure 1F.

Supplementary Figure S6. MagTAQed isolates with altered cellular morphology.

Supplementary Figure S7. Genome rearrangements of MagTAQed isolates.

Supplementary Figure S8. Schematic diagrams of NAHR in the MagTAQed isolates.

Supplementary Figure S9. Cell viability after transient blue light exposure with or without MagMboI-expressing strains.

Supplementary Figure S10. Haploidization in MagTAQed isolate mal61.

Supplementary Figure S11. Meiotic progression of MagTAQed hybrid strains.

Supplementary Figure S12. Random spore analysis of *spo11*Δ hybrid strain expressing MagMboI.

Supplementary Figure S13. Coverage plots of recombined chromosome XV in *SPO11*$^{+}$ spores dissected from a single tetrad.

Supplementary Figure S14. Meiotic recombination in MagTAQed spores.

Supplementary Figure S15. Sequencing coverage of MagTAQed spores.

Supplementary Figure S16. Higher-order chromosome structure in meiosis.

Supplementary Figure S17. Genome rearrangement of MagTAQed RTG mutants.

Supplementary Figure S18. Ectopic translocations in the MagTAQed RTG isolates.

Supplementary tables are in separated Excel files

Supplementary Table S1. Strains used in this study

Supplementary Table S2. Primer sets used to confirm translocations

Supplementary Table S3. Mutations in MagTAQed isolates

Supplementary Table S4. Structural variations in MagTAQed RTG isolates

Supplementary Table S5. Meiotic recombination in *SPO11*$^{+}$ spores

Supplementary Table S6. Meiotic recombination in MagTAQed spores

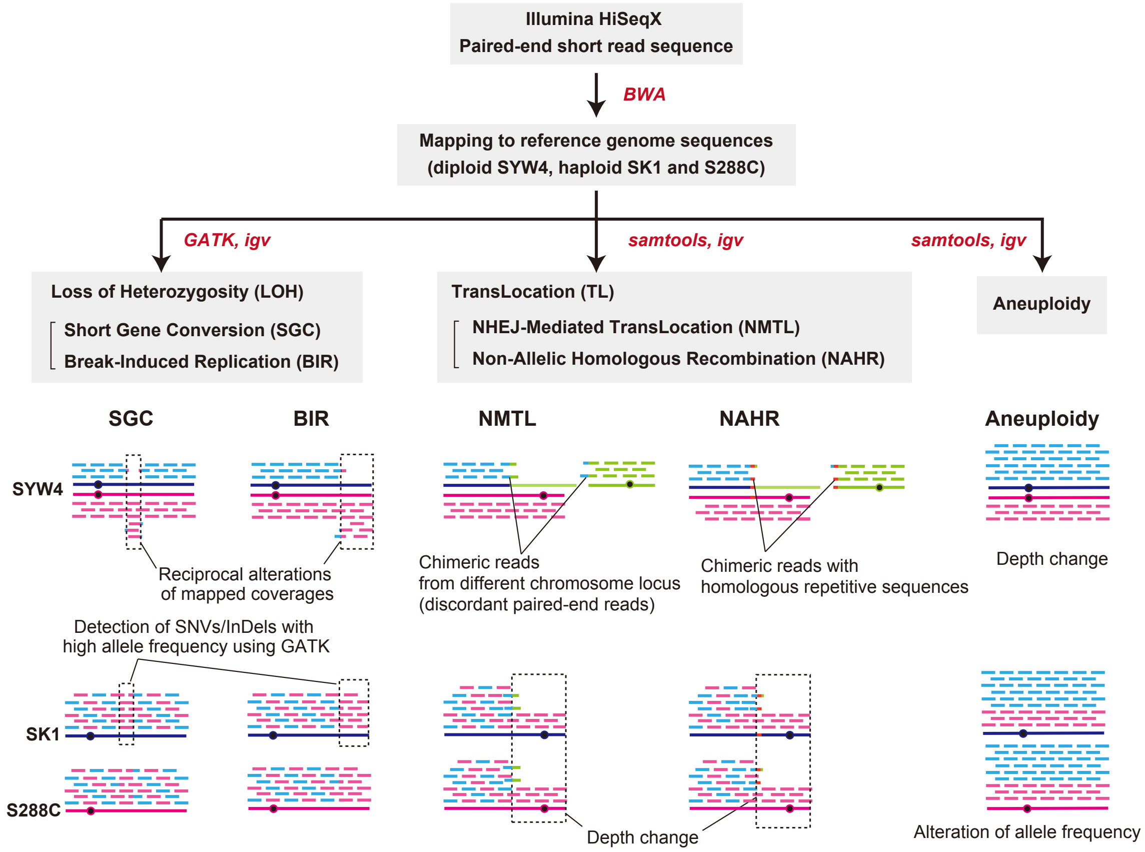

Supplementary Figure S1. Bioinformatic workflow to characterize the mutation events in the MagTAQed diploid strains.

Structural mutations were classified by sequence coverage, read alignment, and allele frequency.

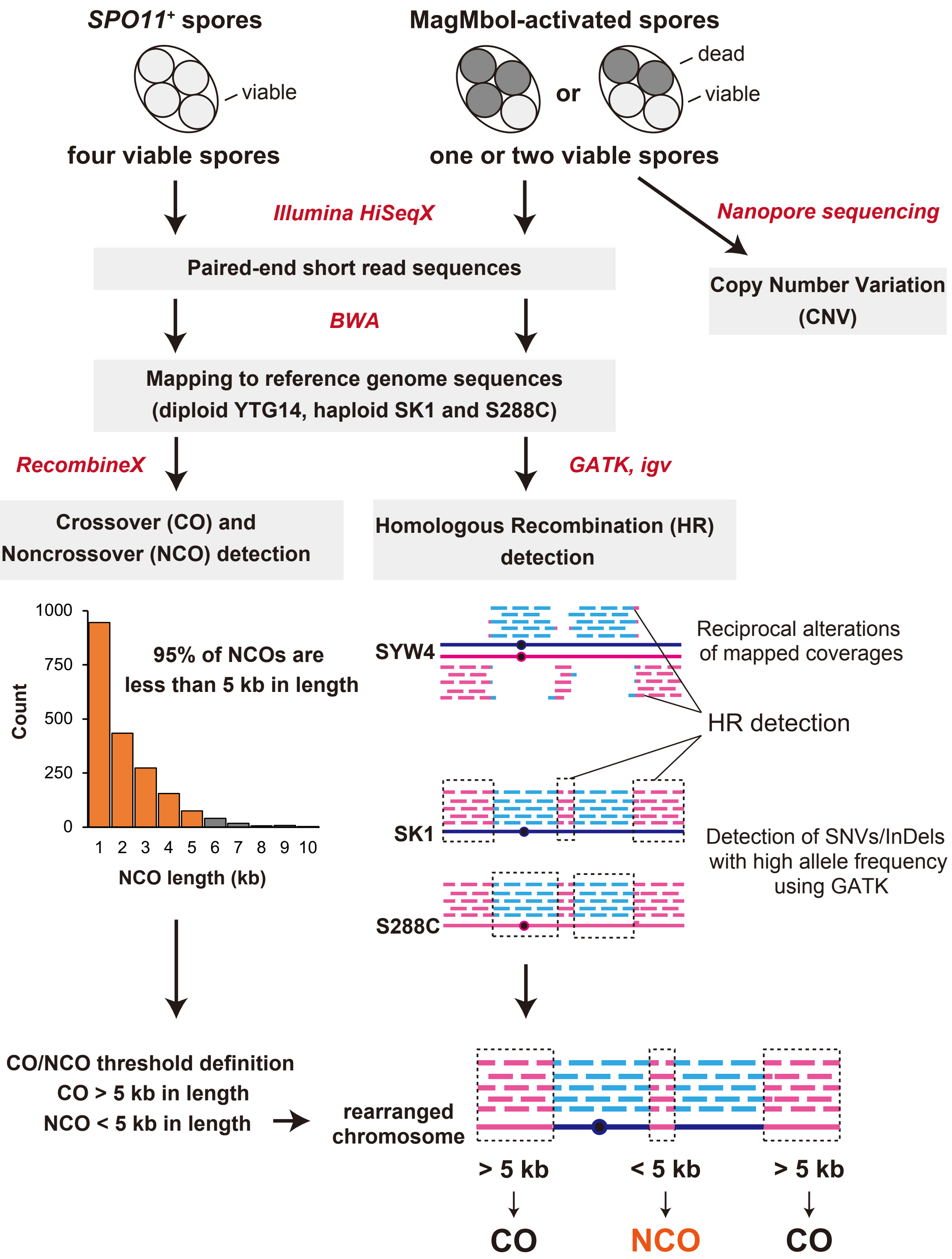

Supplementary Figure S2. Bioinformatic workflow to characterize the mutation events in the MagTAQed haploid spores.

Meiotic recombinations in *SPO11*$^{+}$ spores were detected by RecombineX. Since 95% of the NCOs induced by Spo11 were within 5 kb in length, we classified COs and NCOs induced by MagMboI based on a threshold of 5 kb in interval size.

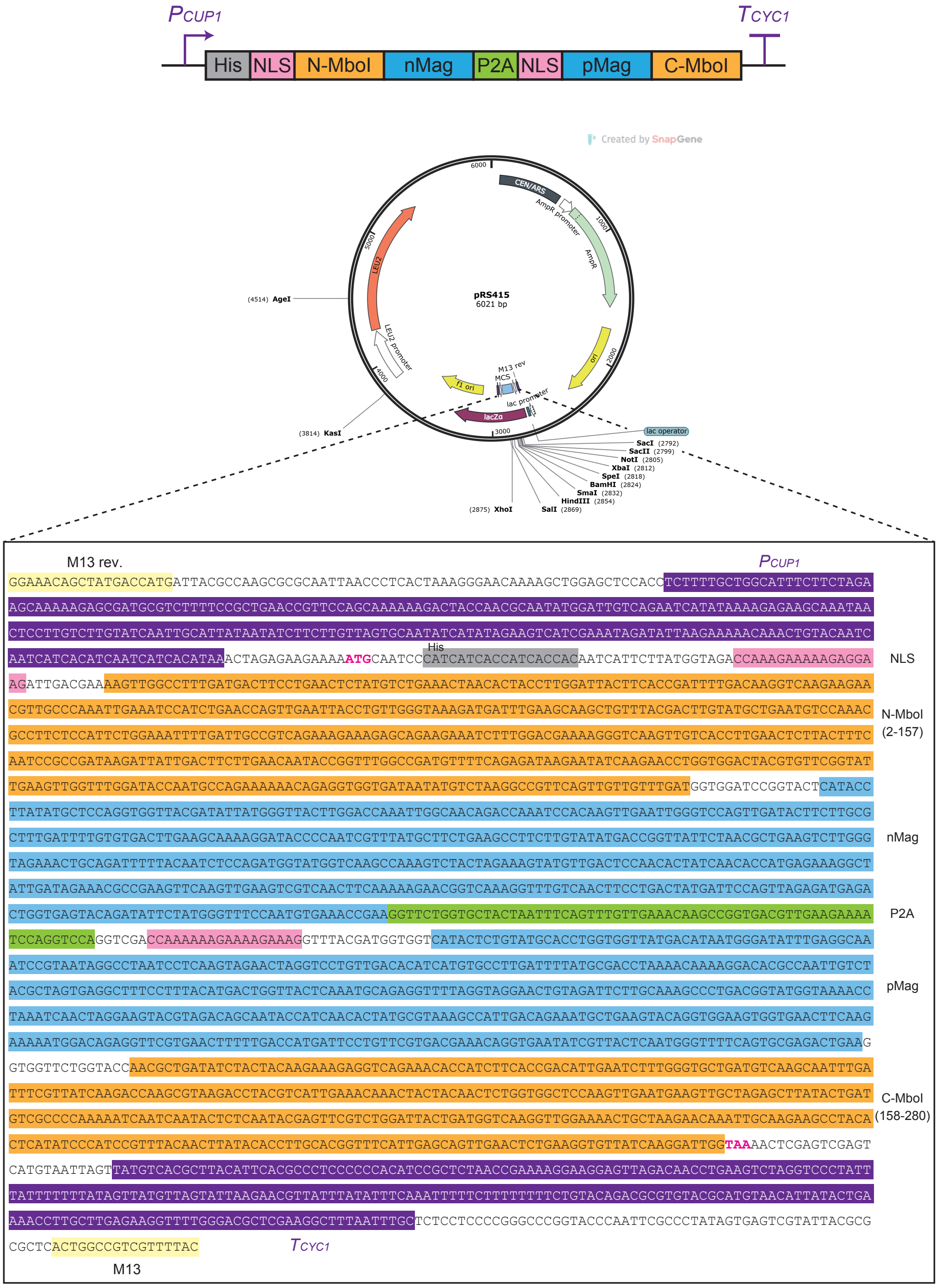

Supplementary Figure S3. Constructs of MagMboI.

(Upper) A schematic diagram of *MagMboI-8* gene. The MboI sequence was split into the N-terminal (N-MboI) and C-terminal (C-MboI) fragments using nMag and pMag. The *CUP1* promoter (P_{CUP1}) and the *CYC1* terminator (T_{CYC1}) were inserted upstream and downstream of the MagMboI gene, respectively. **(Lower)** Nucleic acid sequence of *MagMboI-8* gene cloned into pRS415.

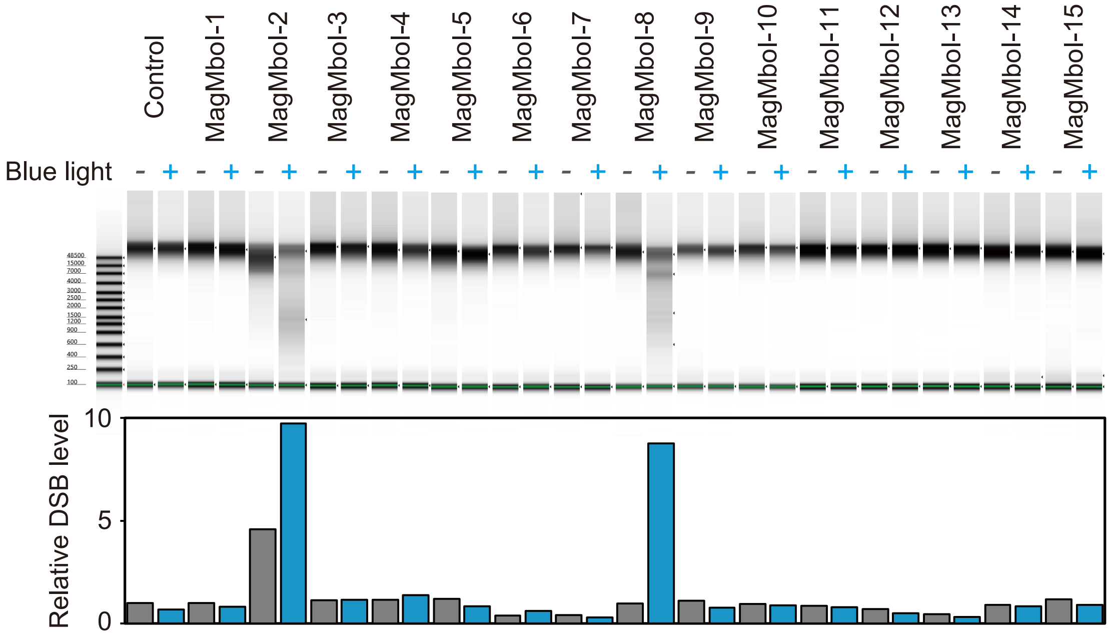

Supplementary Figure S4. Quantification of cleaved DNA for MagMboI variants.

(Upper) The image shows the electrophoresis results analyzed by the TapeStation system (Agilent Technologies) for all 15 MagMboI variants, with or without blue light exposure. **(Lower)** A graph showing quantification of cleaved DNA amounts normalized against the control condition (empty vector) without blue light exposure. This figure includes data of Figure 1D.

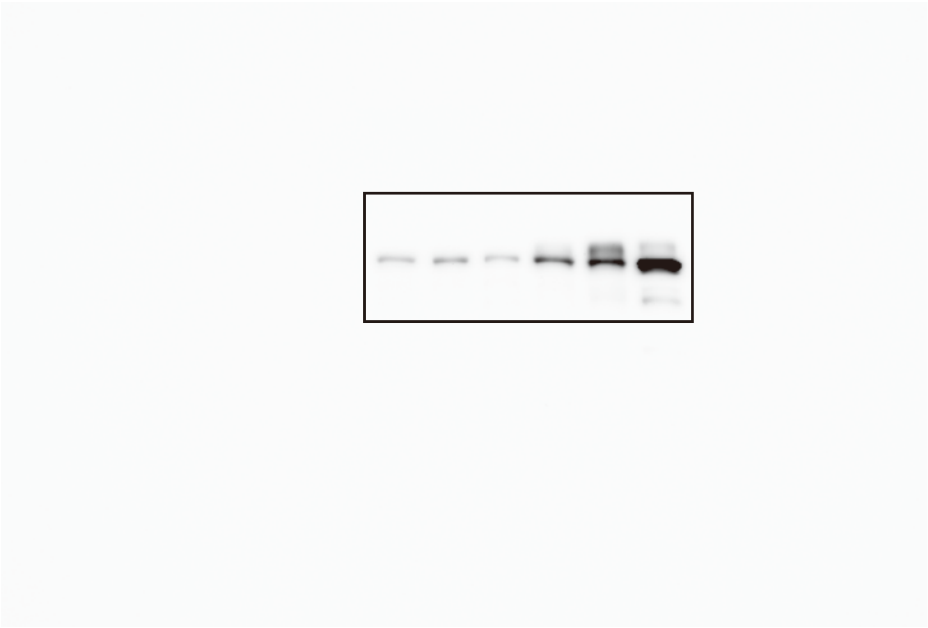

Supplementary Figure 5. Uncropped image used in Figure 1F.
This image has been cropped and shown with different contrast.

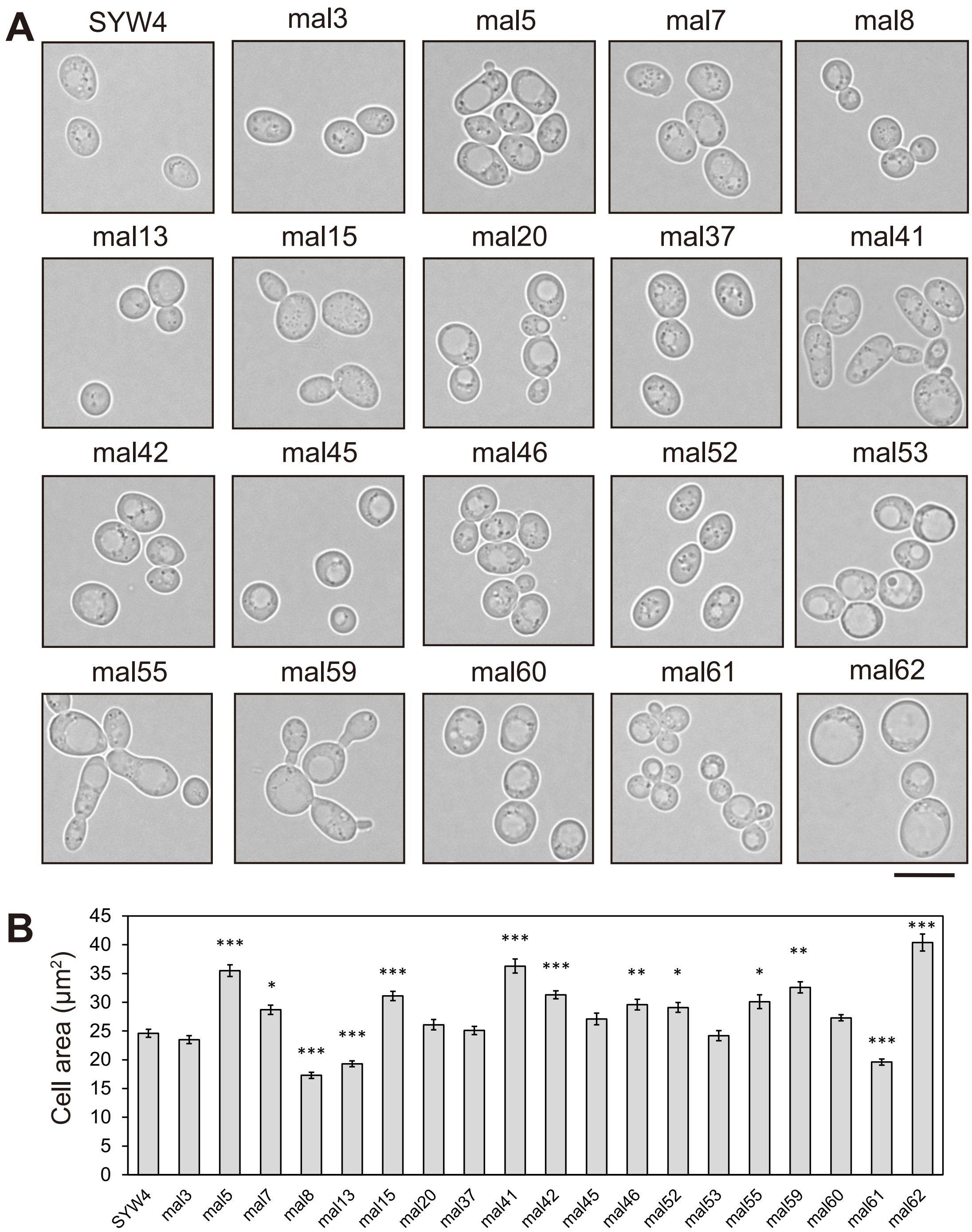

Supplementary Figure S6. MagTAQed isolates with altered cellular morphology. (**A**) Representative images of MagTAQed isolates. Scale bar represents 10 μm. (**B**) Cell areas of MagTAQed isolates. Significant differences were assessed by Dunnett's tests (*, $P < 0.01$; **, $P < 0.001$; ***, $P < 0.0001$). Error bars represent S.E.M (n=50).

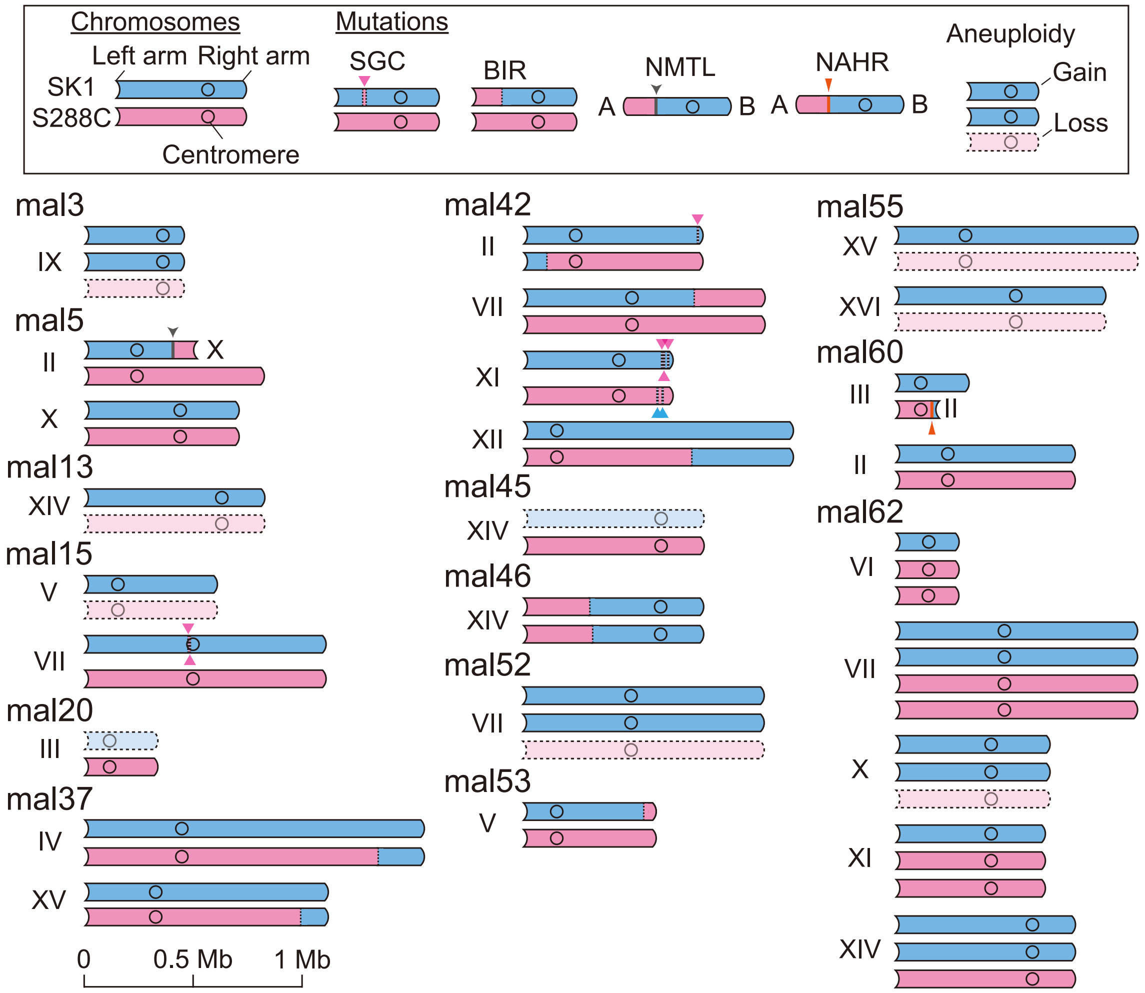

Supplementary Figure S7. Genome rearrangements of MagTAQed isolates.

Schematic diagrams of rearranged chromosomes in MagTAQed isolates.

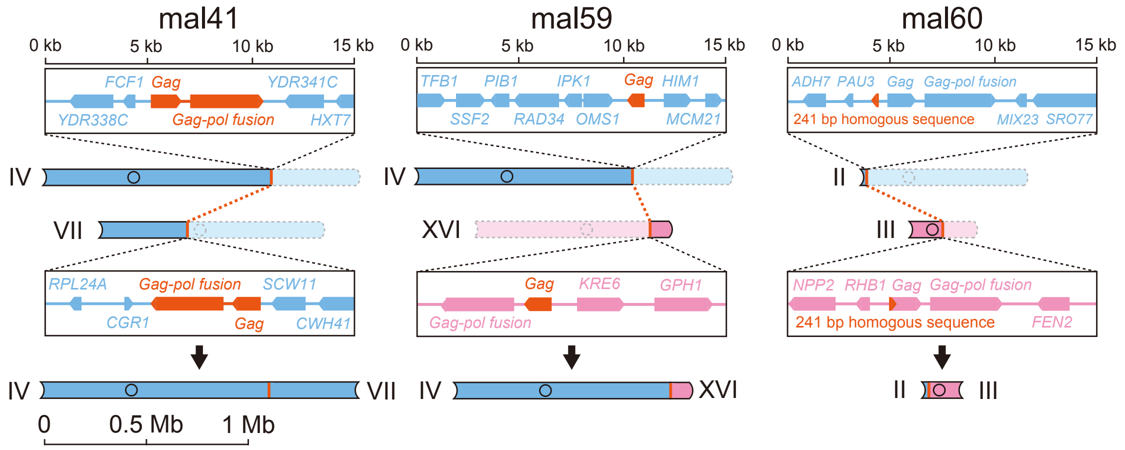

Supplementary Figure S8. Schematic diagrams of NAHR in the MagTAQed isolates.

Magnified views of breakpoints of NAHRs mediated by Ty retrotransposons.

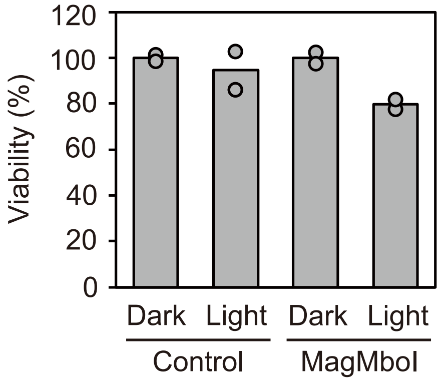

Supplementary Figure S9. Cell viability after transient blue light exposure with or without MagMboI-expressing strains.

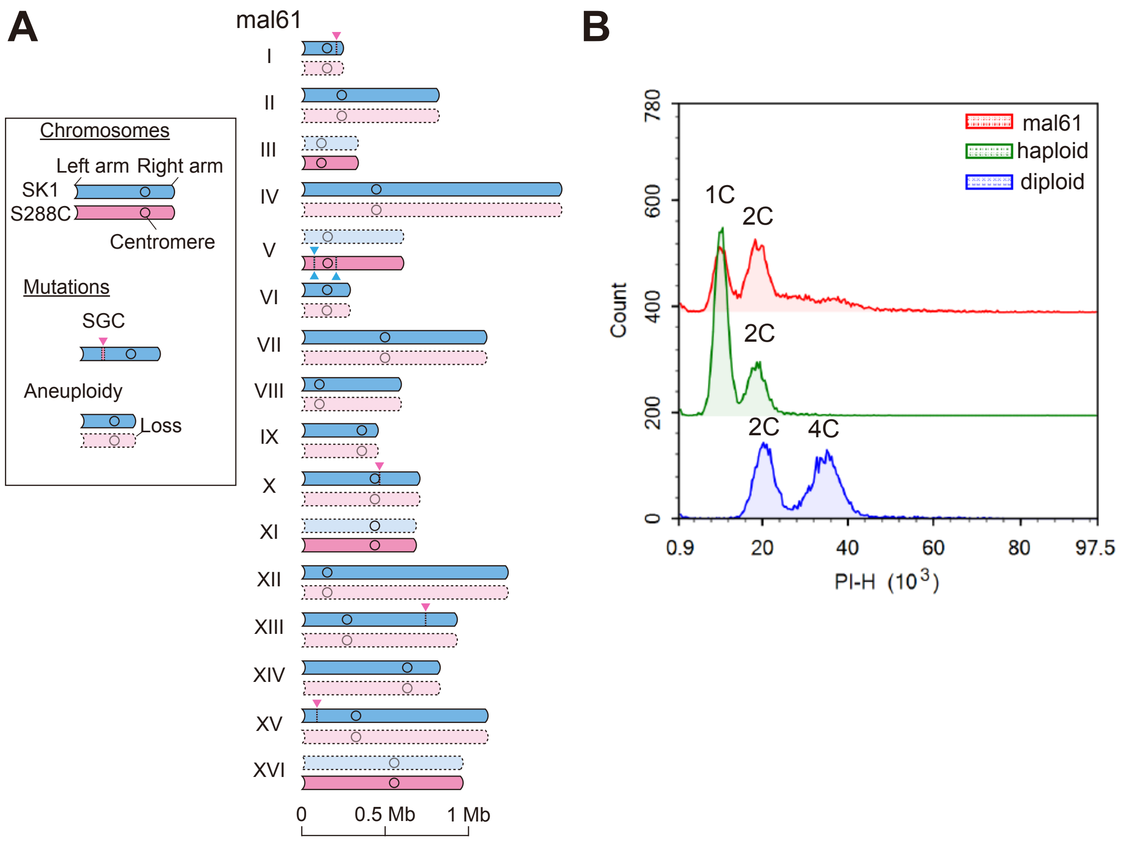

Supplementary Figure S10. Haploidization in MagTAQed isolate mal61.

(**A**) Schematic diagram of chromosomes in the MagTAQed isolate mal61. (**B**) Flow cytometry analysis of ploidy in the mal61, the haploid strain (YPH499), and the diploid strain (SYW4). Each peak indicates a nuclear phase (1C, 2C, and 4C).

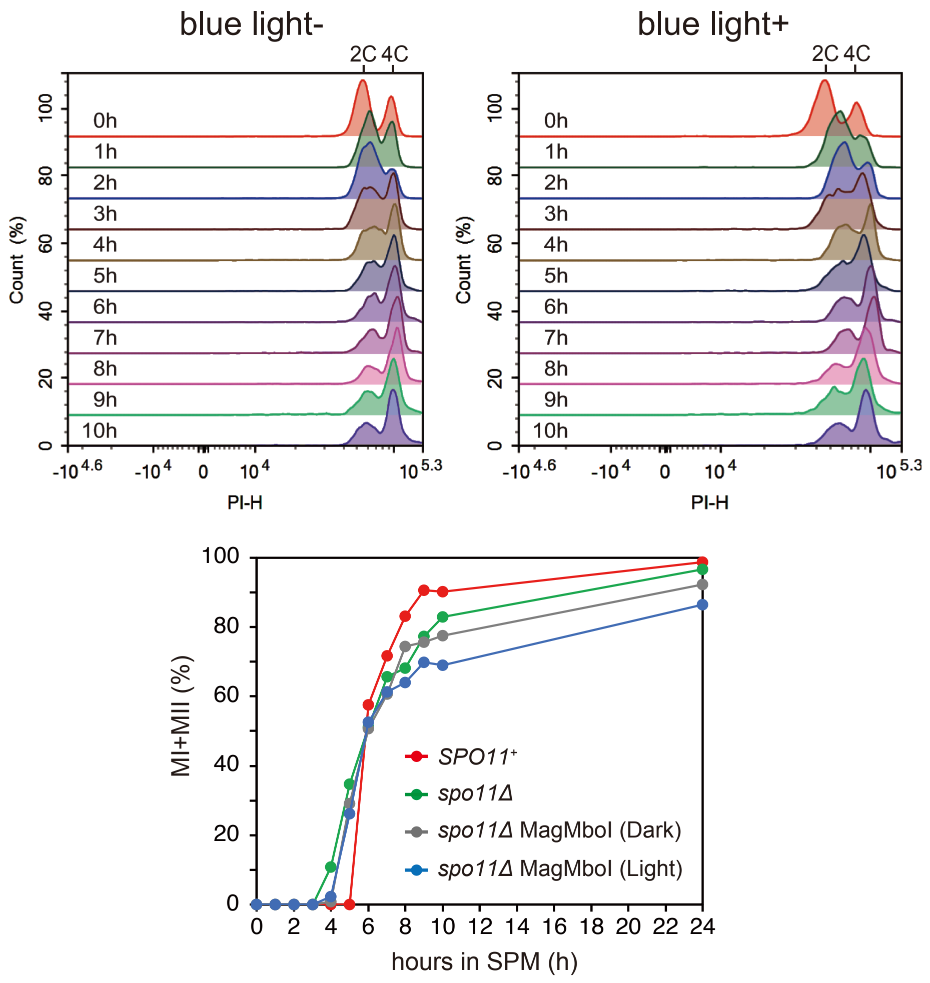

Supplementary Figure S11. Meiotic progression of MagTAQed hybrid strains. **(Upper)** Flow cytometry of propidium iodide (PI)-stained *spo11*Δ strain expressing MagMboI during meiosis (0-10 hours). Flow cytometry histograms indicate cell counts. 2C or 4C represents two or four copies of the genomic DNA. **(Lower)** Meiotic divisions of DAPI-stained *spo11*Δ cells expressing MagMboI were analyzed by microscopic observation. The proportion of cells that completed meiosis I (MI) and meiosis II (MII) at indicated time points (MI + MII) are shown. Cells were exposed to blue light for 30 min after 3.5 hours culture in SPM medium. More than 150 cells were counted at each time point. Data of *SPO11*$^{+}$ and *spo11*Δ are from Kawashima et al., 2023 (42). The values of *spo11*Δ MagMboI (Dark) and *spo11*Δ MagMboI (Light) represent mean values (*n*=2).

Spore viability (%)

Genotype	Dark	Blue light
spo11Δ	0.13	0.15
spo11Δ MagMboI	0.25	0.37

Supplementary Figure S12. Random spore analysis of *spo11*Δ hybrid strain expressing MagMboI.

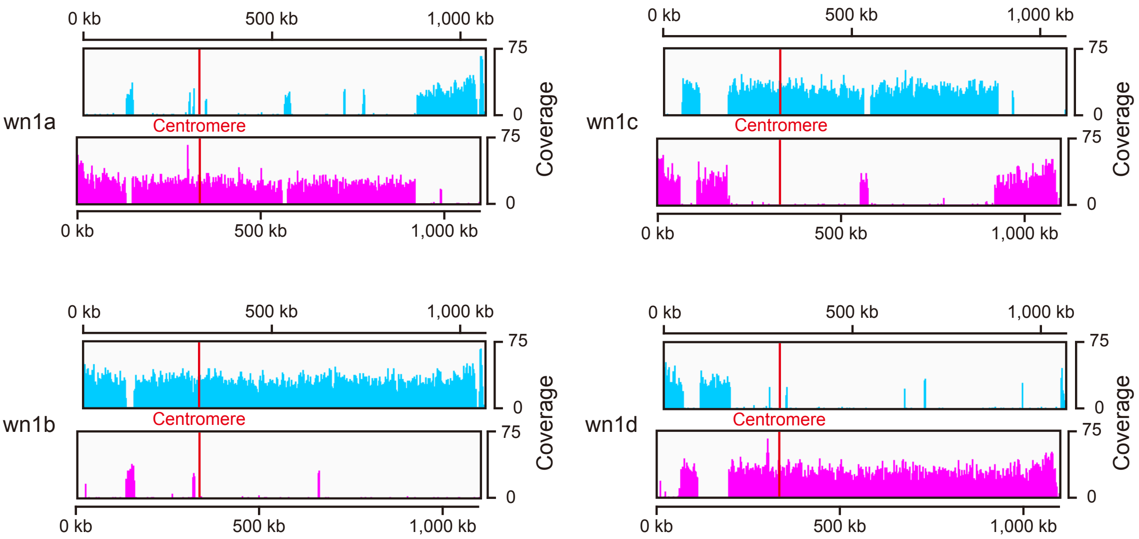

Supplementary Figure S13. Coverage plots of recombined chromosome XV in *SPO11*$^{+}$ spores dissected from a single tetrad.

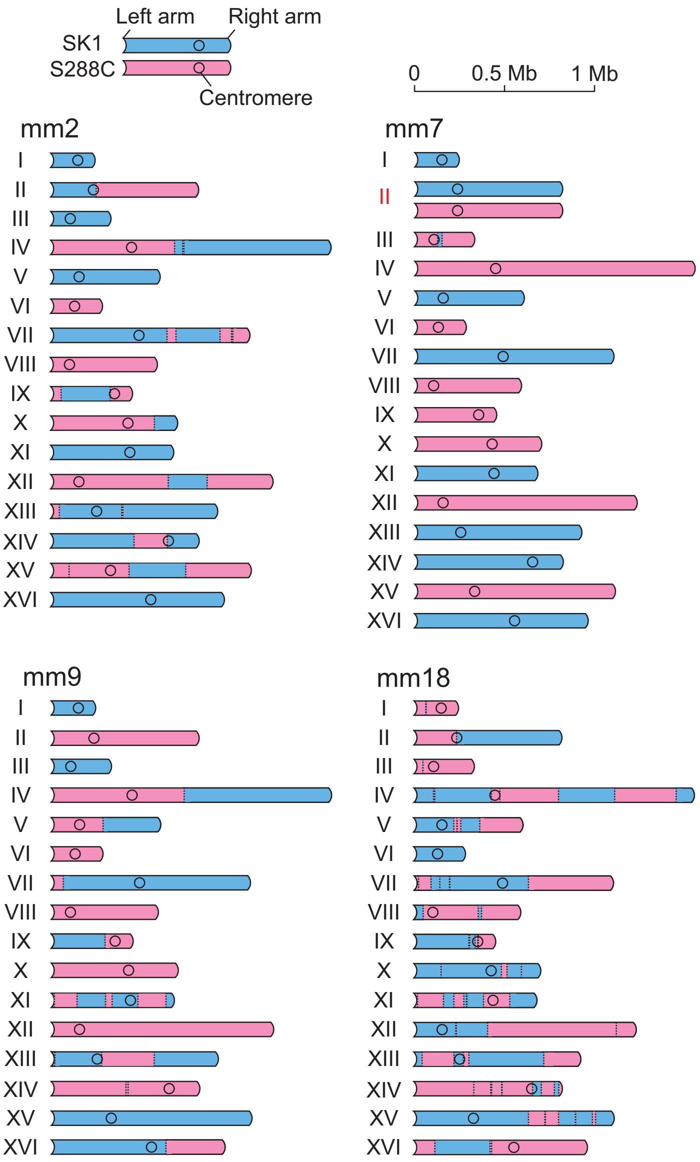

Supplementary Figure S14. Meiotic recombination in MagTAQed spores.

Schematic diagrams of recombined chromosomes in MagTAQed spores. The name of aneuploid chromosome is shown in red type.

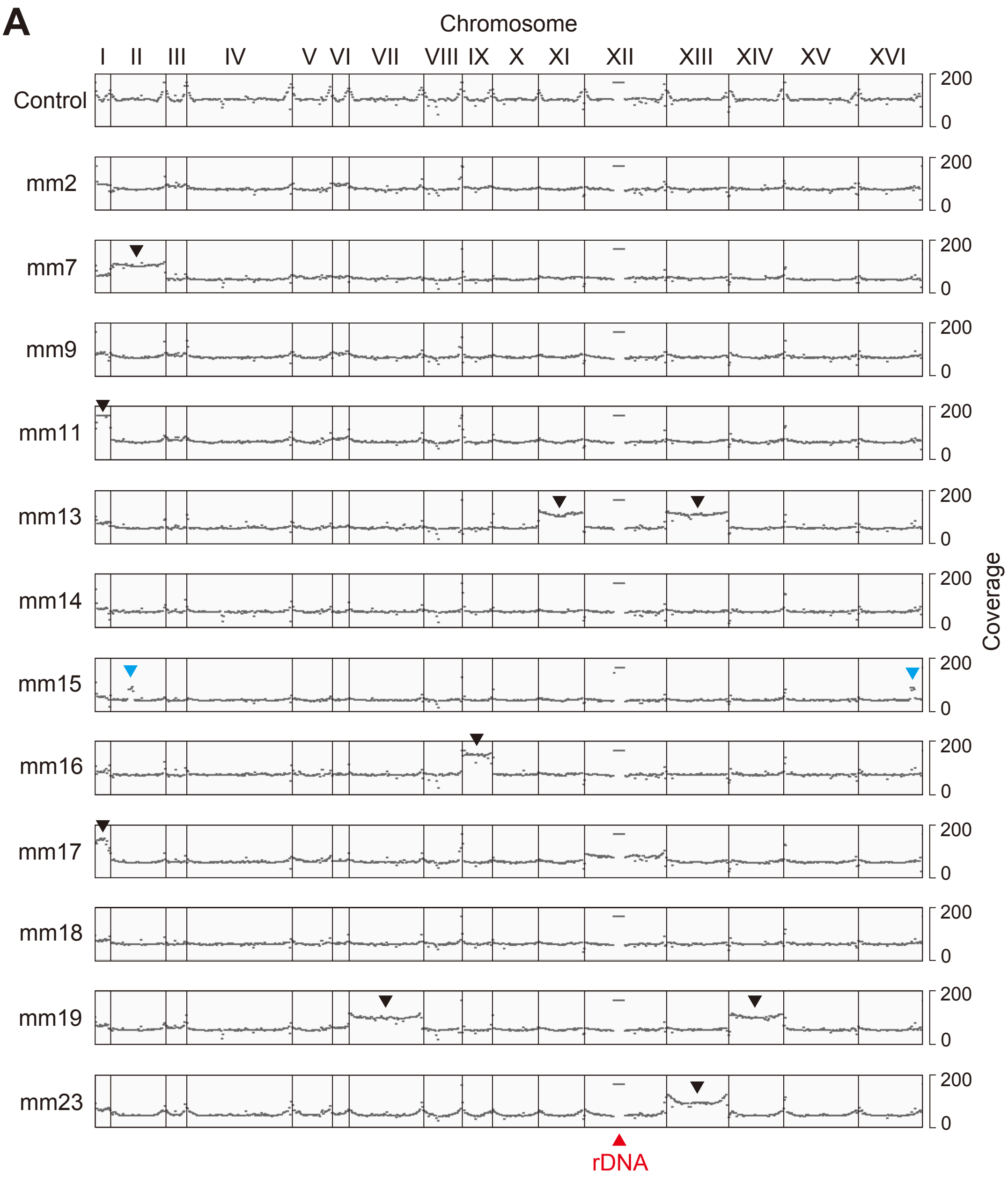
A
Chromosome
I II III IV V VI VII VIII IX X XI XII XIII XIV XV XVI
Control
mm2
mm7
mm9
mm11
mm13
mm14
mm15
mm16
mm17
mm18
mm19
mm23
200
0
Coverage
rDNA

B

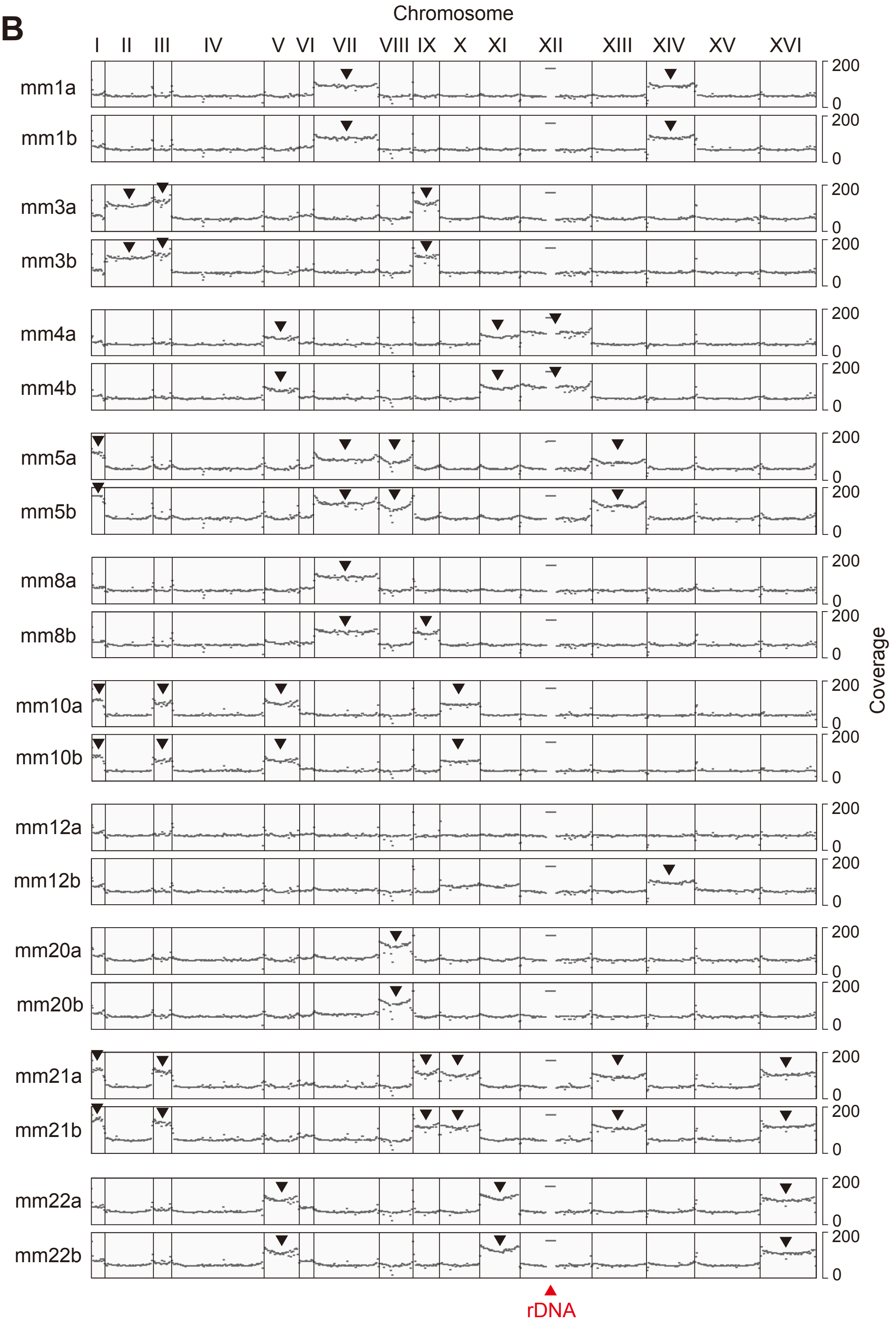
Chromosome
I II III IV V VI VII VIII IX X XI XII XIII XIV XV XVI
mm1a
mm1b
mm3a
mm3b
mm4a
mm4b
mm5a
mm5b
mm8a
mm8b
mm10a
mm10b
mm12a
mm12b
mm20a
mm20b
mm21a
mm21b
mm22a
mm22b
200
0
Coverage
rDNA

Supplementary Figure S15. Sequencing coverage of MagTAQed spores.

Read depth of (**A**) one viable spore and (**B**) two viable spores from a tetrad. Black arrowheads indicate aneuploid chromosomes. Red arrowheads indicate rDNA regions in chromosome XII. Blue arrowheads indicate copy number variations.

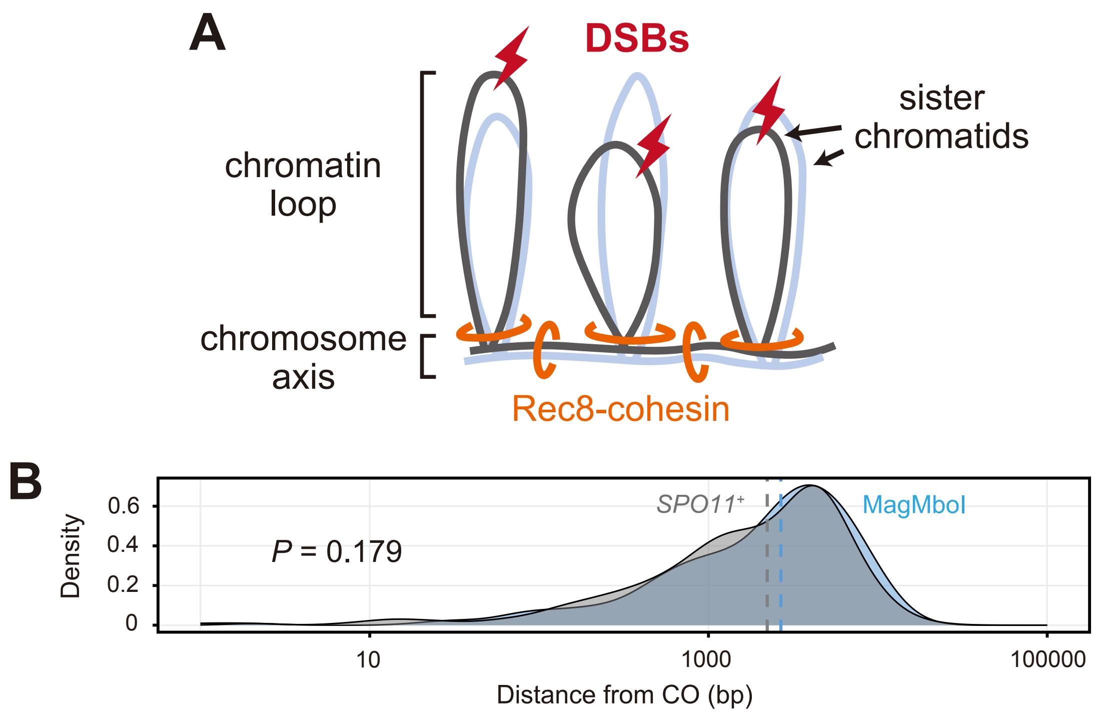

Supplementary Figure S16. Higher-order chromosome structure in meiosis.
(**A**) Chromosome axis and meiotic DSBs. Meiosis-specific cohesin Rec8 is required for sister chromatid cohesion and chromosome axis formation. Spo11-induced DSBs are preferentially formed within the chromatin loops. (**B**) Distance from COs to Rec8 binding sites in the *SPO11*$^{+}$ (39) and MagTAQed spores. The Rec8 binding sites were identified by chromatin immunoprecipitation sequencing (28).

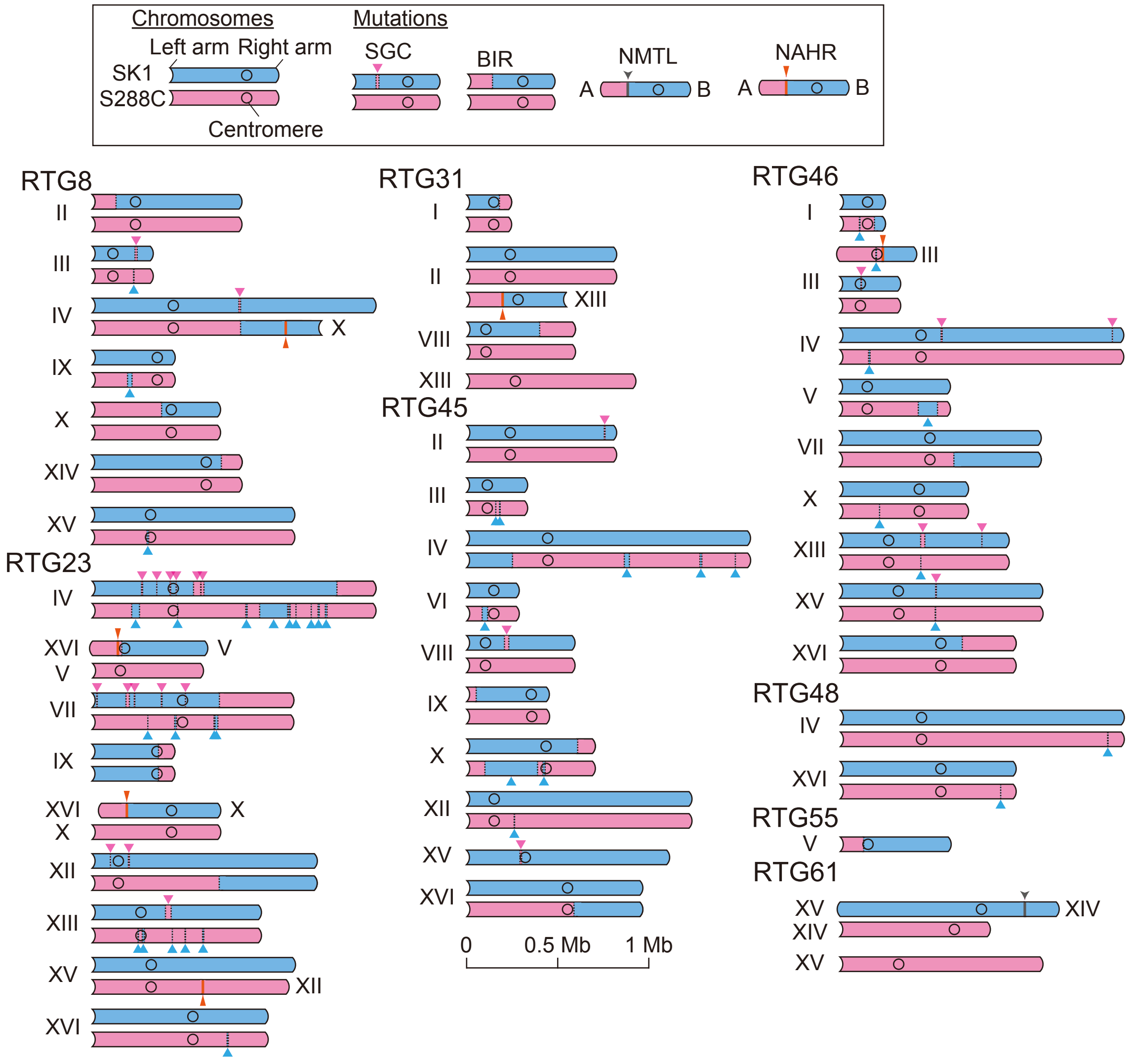

Supplementary Figure S17. Genome rearrangement of MagTAQed RTG mutants.
Schematic diagrams of recombined chromosomes with in MagTAQed RTG mutants.

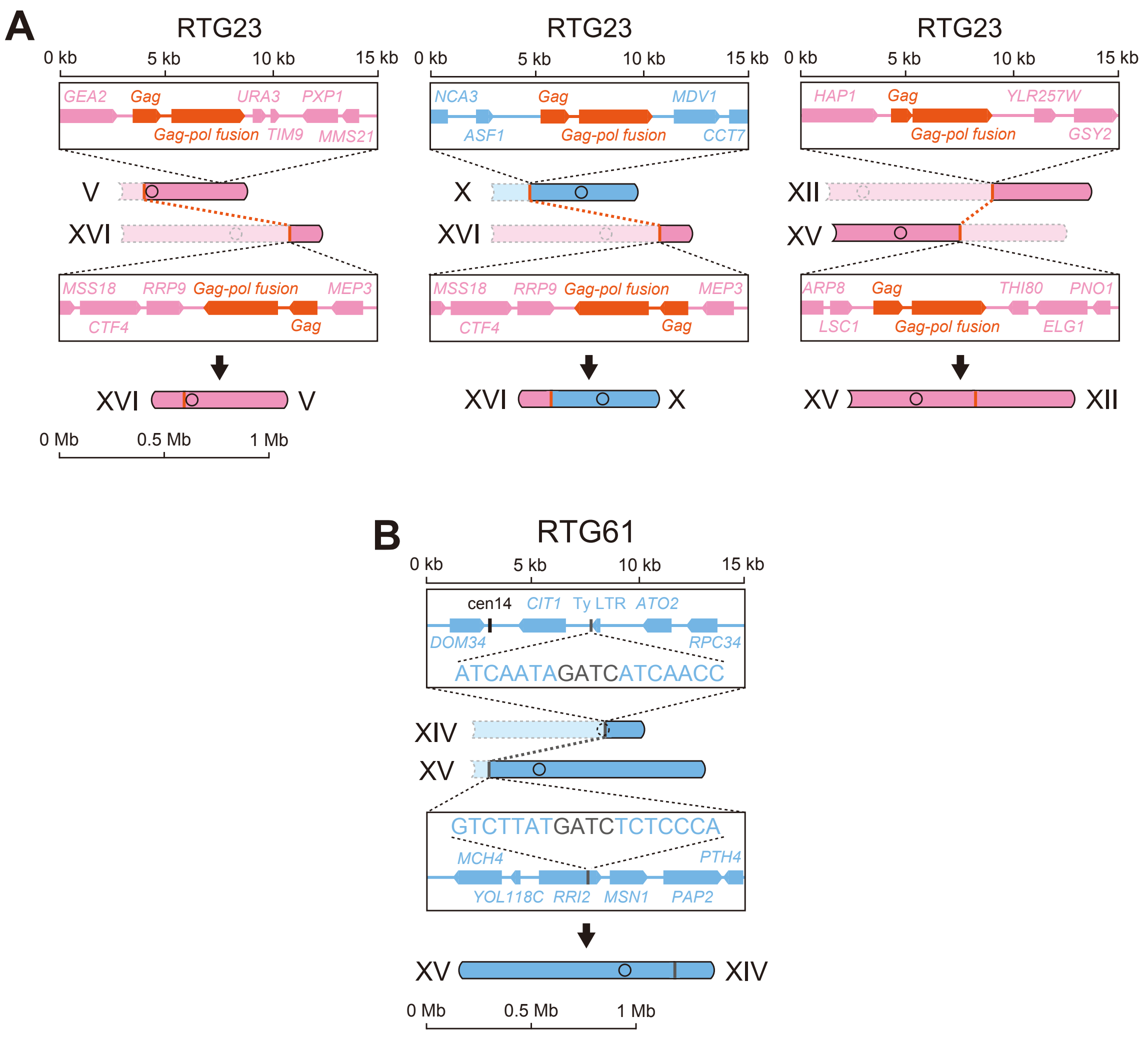

Supplementary Figure S18. Ectopic translocations in the MagTAQed RTG isolates. Magnified views of breakpoints of (**A**) NAHRs mediated by Ty retrotransposons and (**B**) NMTL mediated by MboI recognition sites.
